# How the biomimetic assembly of membrane receptors into multivalent domains is regulated by a small ligand†

**DOI:** 10.1039/d1sc01598b

**Published:** 2021-04-30

**Authors:** Anna Grochmal, Ben Woods, Lilia Milanesi, Manuel Perez-Soto, Salvador Tomas

**Affiliations:** Department of Biological Sciences, Institute of Structural and Molecular Biology, School of Science, Birkbeck University of London Malet Street London WC1E 7HX UK s.tomas@bbk.ac.uk

## Abstract

In living cells, communication requires the action of membrane receptors that are activated following very small environmental changes. A binary all-or-nothing behavior follows, making the organism extremely efficient at responding to specific stimuli. Using a minimal system composed of lipid vesicles, chemical models of a membrane receptor and their ligands, we show that bio-mimetic ON/OFF assembly of high avidity, multivalent domains is triggered by small temperature changes. Moreover, the intensity of the ON signal at the onset of the switch is modulated by the presence of small, weakly binding divalent ligands, reminiscent of the action of primary messengers in biological systems. Based on the analysis of spectroscopic data, we develop a mathematical model that rigorously describes the temperature-dependent switching of the membrane receptor assembly and ligand binding. From this we derive an equation that predicts the intensity of the modulation of the ON signal by the ligand-messenger as a function of the pairwise binding parameters, the number of binding sites that it features and the concentration. The behavior of our system, and the model derived, highlight the usefulness of weakly binding ligands in the regulation of membrane receptors and the pitfalls inherent to their binding promiscuity, such as non-specific binding to the membrane. Our model, and the equations derived from it, offer a valuable tool for the study of membrane receptors in both biological and biomimetic settings. The latter can be exploited to program membrane receptor avidity on sensing vesicles, create hierarchical protocell tissues or develop highly specific drug delivery vehicles.

## Abstract 2

In lipid vesicles near their membrane phase-transition temperature, the presence of a small, weakly binding ligand tips the balance for the assembly of multivalent receptor domains. We recapitulate this behaviour using a global binding-clustering model.

## Graphical abstract

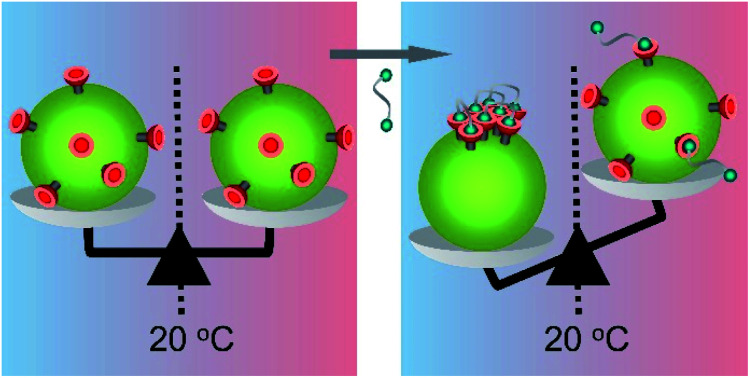

## 1. Introduction

Receptors located on the cell membrane bind to ligands present in solution or displayed on other surfaces, and play a central role in cell adhesion and communication processes.^1–3^ The regulation of receptors involves the participation of complex machinery, tasked with the detection of the appropriate environmental changes and activation of the receptors (for example, by recruiting them at the point of interaction into platforms of high avidity) when the level of a given stimulus reaches a critical threshold.^2^ Identifying the switches that control the function of these receptors is a very challenging task in the context of the biomolecular complexity of the cell.^4–6^ Simple models of a cell membrane, in the form of lipid vesicles equipped with minimal synthetic membrane receptors and ligands, have shown that the lateral assembly of receptors enhances both binding to ligands in solution^7,8^ and membrane adhesion.^9,10^ These studies have shown that large increases in avidity are largely due to a multivalent effect. The corollary is that ON/OFF regulation of membrane receptor function can be achieved by means of a bimodal control of the receptor assembly, *i.e.* non-assembled monovalent state (low avidity, OFF state) *versus* assembled multivalent platforms (high avidity, ON state). On the one hand, identifying and characterizing stimuli that lead to extensive lateral assembly of membrane receptors will improve our understanding of cell communication processes.^11^ On the other hand, it will inform the design of minimal protocells that are capable of communicating with each other^12,13^ or to adhere to living cells upon stimulus.^14,15^ The latter will also facilitate the development of efficient vehicles for targeted drug delivery.

In our earlier work, we rigorously characterized the mutual modulation between the lateral assembly of membrane receptors and the binding of ligands in solution.^7,16^ The mathematical model we developed allowed us to quantify the enhancement of binding due to the formation of multivalent receptor clusters and relate it to a multivalent effect. In the present work we show that changes in the membrane phase (from liquid disordered to gel) lead to an ON/OFF bimodal response in the assembly of the membrane embedded receptor. Moreover, we show that ligands in solution modulate the intensity of the ON response of the bimodal switch, and that efficient modulation of the signal (from virtually non-existent to a maximum response where most of the receptors present in the membrane are assembled) does not require strong ligand binding. We have developed a mathematical assembly-binding model that uses a van't Hoff approach to account for the temperature dependence of the assembly. The fit of this model to UV-visible spectroscopy titration data at different temperatures is excellent, and tracks changes consistent with temperature-induced receptor assembly, ligand induced binding and assembly (or disassembly, when a large excess of ligand is added). These results and the model derived highlight that the bimodal assembly is driven largely by the dramatic increase in lipid–lipid interactions upon phase transition, while ligand binding leads to small changes in receptor assembly that, in the appropriate conditions, are enough to tip the system towards extensive receptor assembly. We reason that any stimulus capable of modulating lipid–lipid interactions (in this instance temperature, but reasonably also lipophilic drugs or hormones) has the potential to trigger this type of switch. The described phenomenon could be programmed into vesicle-based protocells, leading to biomimetic specificity and control of ligand binding and membrane adhesion.

## 2. Results and discussion

### 2.1. Lateral assembly of the receptor

**Fig. 1:**
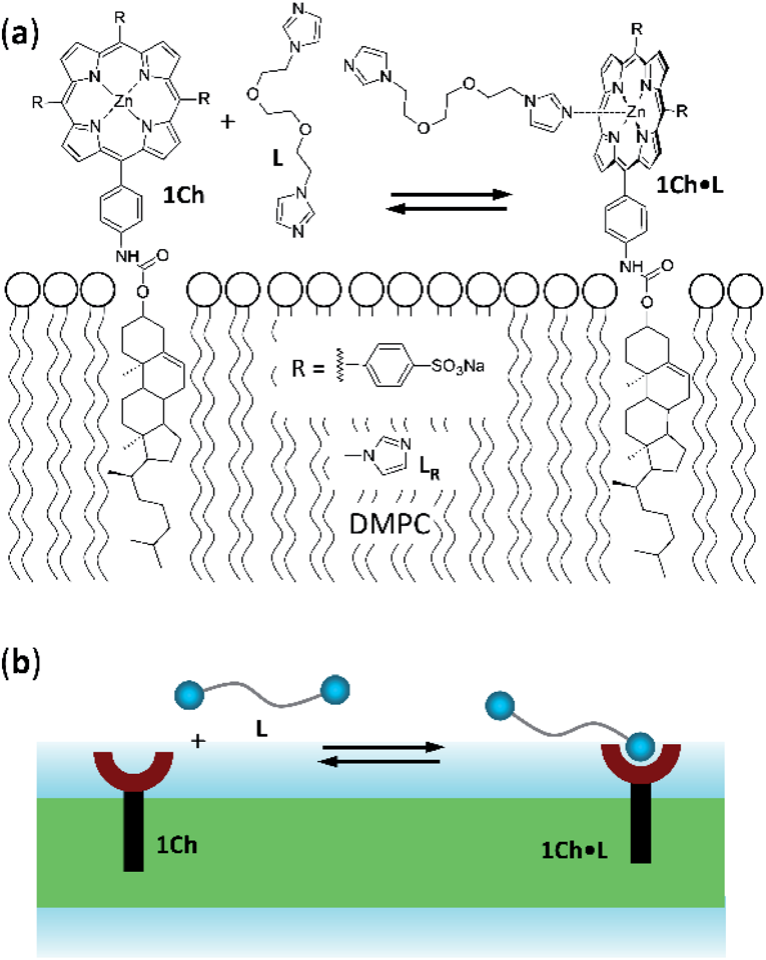
(a) Chemical structures of receptor **1Ch**, ligands **L** and **LR** and complex **1Ch·L** represented embedded in a DMPC membrane. (b) Cartoon representation of the structures shown in (a).

Receptor **1Ch** (Fig. 1) contains a chromophoric Zn metalloporphyrin headgroup, which allows the monitoring of the lateral assembly and the binding of amine-containing ligands using UV-visible spectroscopy. A cholesterol membrane anchor ensures efficient insertion of **1Ch** into the membrane of lipid vesicles.^7,16^ In our earlier work, we have shown that increasing the in-membrane concentration of **1Ch** in lipid vesicles (membrane composition DMPC/cholesterol 80 : 20 mol/mol) leads to a red-shift of the Soret band of the porphyrin moieties UV-visible spectrum, characteristic of J-aggregates.^17^ These changes are attributed to the lateral assembly of **1Ch** into domains, or clusters C.

**Fig. 2:**
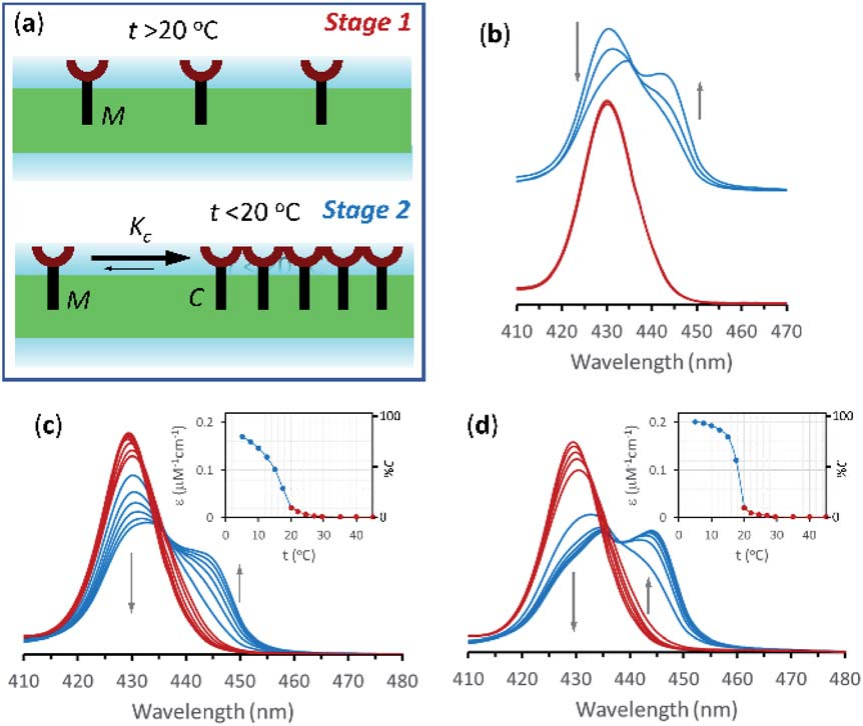
(a) Cartoon representation illustrating the absence of lateral assembly at high temperature (top) and of a nucleation-growth mechanisms of lateral assembly of **1Ch** in the membrane at low temperature (bottom). (b) Soret band region of selected UV-visible spectra of membrane embedded **1Ch** with an in-membrane concentration *r***1Ch** = 0.01, 0.015 and 0.025. The red spectra (bottom traces) were recorded at 22 °C and the blue spectra (top traces) at 15 °C. The grey arrows indicate the direction of change as the in-membrane concentration is increased. (c). Changes in the Soret band region of the UV-visible spectra of membrane embedded **1Ch** (*r***1Ch** = 0.01) with changes in temperature (from 50 °C to 5 °C). The grey arrows indicate the direction of change as the temperature decreases. The red spectra are recorded at temperatures ≥ 20 °C and the blue ones below 20 °C. Inset: changes in the apparent extinction coefficient at 445 nm (solid circles). The joining line is a visual aid. The right-hand axis shows the percentage of the clustered form C in relation to total **1Ch**, calculated from the *r*_M,max_ at the corresponding temperature. (d) Idem, for *r***1Ch** = 0.025.

In the current work we use vesicles composed of pure DMPC. Anchored in the membrane of these vesicles, **1Ch** does not show any discernible change in the UV-visible spectrum above 20 °C (henceforth temperature stage 1, Fig. 2a) and up to in-membrane concentrations (termed here *r***1Ch**, measured as the molar ratio of **1Ch** over that of lipids) of 0.025. This result is consistent with a negligible lateral assembly of the monomeric form of the receptor **1Ch**, which we term M, into clustered form C, within this temperature and concentration range (Fig. 2b). However, below 20 °C (henceforth temperature stage 2. Fig. 2a) there is a clear growth of the red-shifted Soret band, indicative of the lateral assembly leading to the clustered form of the receptor C.^7,16^

The dependence of the spectrum with the temperature was tested with vesicles at constant in-membrane concentration, (*r***1Ch** = 0.01 and 0.025; Fig. 2c and d, respectively). In these conditions the UV-visible spectra initially experience small but clear temperature-dependent changes when cooled from 30 °C to 20 °C. Since the UV-visible spectrum is concentration-independent in this temperature range (Fig. 2b), we attribute these minor changes to variations in the physical properties of the lipid interface leading to a solvatochromic shift of the M form of the receptor as we approach the phase transition temperature.^18–20^

Below 20 °C (temperature stage 2), the redshifted band, attributed to the laterally assembled C form of **1Ch**, grows rapidly when the temperature decreases, which is steeper in samples with a higher in-membrane concentration of **1Ch** (Fig. 2c and d). These changes are consistent with a cooperative nucleation-growth mechanism for the assembly of the clusters.^21^ This behaviour of the receptor is in contrast with our earlier work, where **1Ch** was shown to undergo lateral assembly in a weak, non-cooperative isodesmic fashion. However, the earlier work focused on the assembly of the receptor at moderately high temperatures (37 °C), higher receptor loadings (with *r***1Ch** up to 0.1) and different membrane composition.^7,16^ Nucleation-growth assembly in the membrane can be characterized by the in-membrane solubility of the monomeric form M of the receptor, *r*_M,max_, or its reciprocal, the clustering constant *K*_c_:1
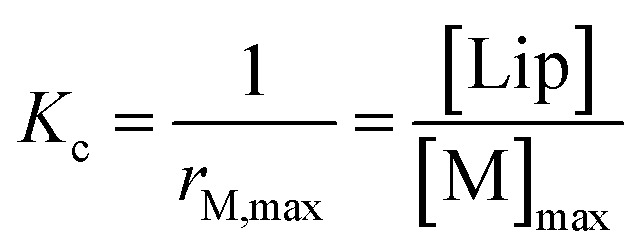


In eqn (1), [M]_max_ and [Lip] are the maximum concentration of the monomeric form of the receptor and that of lipids in relation to the total solution volume. The rise of the red-shifted Soret band (centred at 445 nm *c.a.*), characteristic of the clustered form C (Fig. 2),^7,16^ allows us to determine *r*_M,max_ and *K*_c_ at specific temperatures, during temperature stage 2 (*i.e.*, below 20 °C) (See ESI† for details of the model, ESI Fig. S1 and ESI Table S1†). *r*_M,max_ has a value of 0.007 at 20 °C, (*K*_c_ = 140) and reaches values as low as 0.0027 at 5 °C (*K*_c_ = 370).

### 2.2. Ligand binding

As we have previously reported, the binding of a ligand to a membrane-anchored receptor and the lateral assembly of the receptor modulate each other.^7,16^ The extent of the modulating effect is quantified by the modulation factor, *M*_f_, which is the factor by which the binding affinity for the ligand increases upon lateral assembly, or, conversely, the factor by which the lateral assembly increases upon binding of the ligand. For monovalent ligands the modulation has been attributed to the increased hydrophobicity in the cluster environment, as well as to favourable secondary interactions (*e.g.*, CH–π) between the porphyrin-bound ligand and nearby porphyrins in the cluster.^7^ It is worth considering that ligand binding may disrupt somewhat porphyrin J-aggregates in the cluster. This may counteract somewhat the benefits of binding to the cluster, leading to the observed weak modulation effect. For divalent ligands the overall modulation is a function of the *M*_f_ of each binding and the chelate effect which can lead to a much stronger modulation.^7,16^

For ligand **L** the binding sites are *N*-alkyl imidazole moieties (Fig. 1a). Chemically equivalent monovalent ligand methylimidazole **LR** was used to determine the contribution to receptor binding of each binding site (that is, the microscopic binding constant *K*_m_) and the modulation factor *M*_f_ per binding site at play in divalent **L**. We determined the binding affinity of **LR** for **1Ch** by means of a UV-visible titration method, in experimental conditions where either monomeric form of the receptor, M, or the assembled C form are dominant. From these experiments we obtained a value for the binding constant of **LR** to M, termed, *K*_m_, of 38 M^−1^, which is constant with the temperature within the error of the measure. The modulation factor *M*_f_ obtained was 2.2. (see ESI for details of the model used, ESI Fig S2 and ESI Table S2†). Both values are consistent with those obtained in our previous work.^16^

**Fig. 3:**
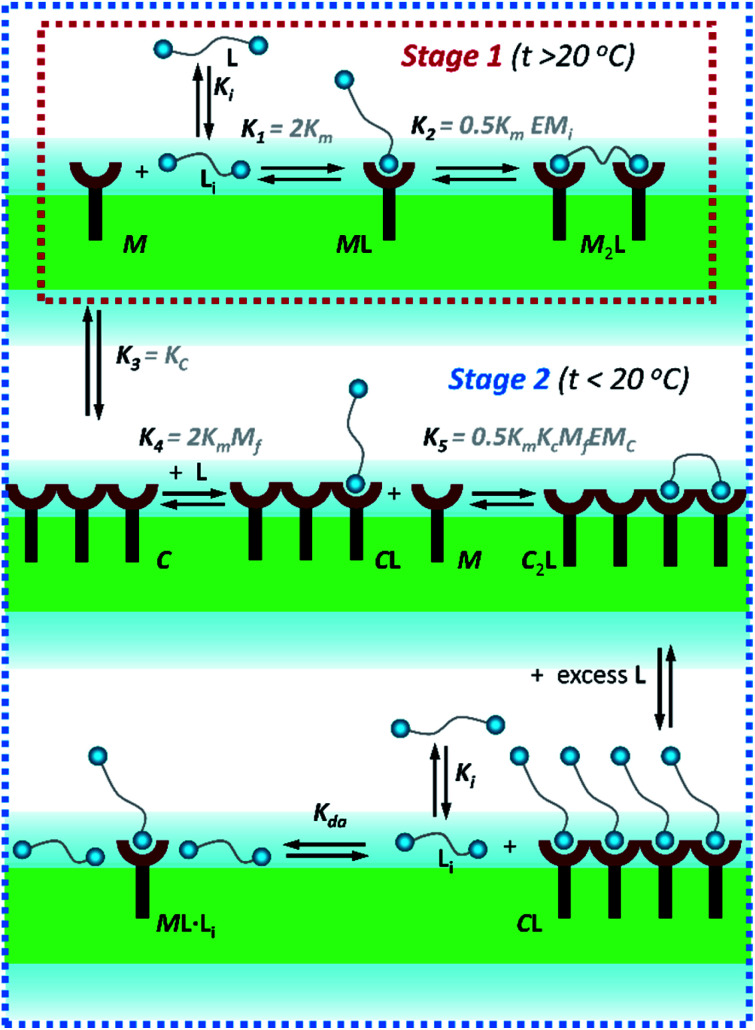
Summary of the main binding and lateral assembly events that dominate each of the temperature stages for membrane embedded receptor **1Ch**. The relevant equilibrium constants are written as a function of the pairwise binding parameters. Only one of the possible routes that lead to C_2_**L** is shown. See ESI Fig. S4† for an extended illustration of the equilibria involved and their relationship with the pairwise binding parameters.

**Fig. 4:**
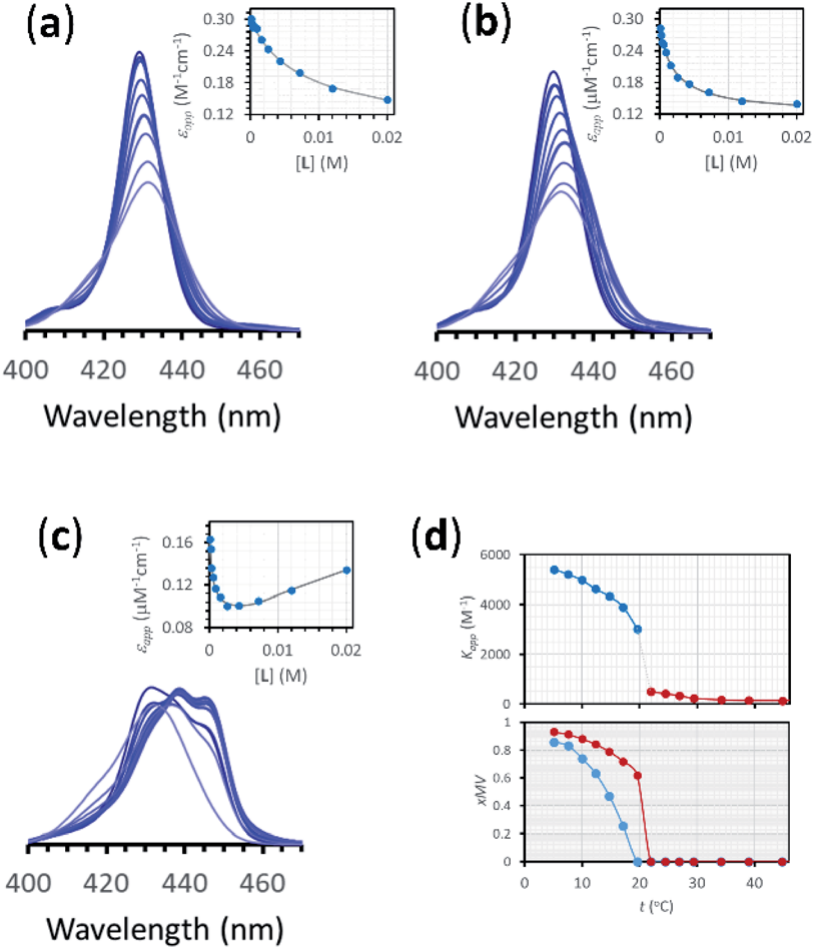
(a) Changes in the Soret band region of the UV-visible spectrum of membrane embedded **1Ch** upon addition of increasing amounts of ligand **L** at 45 °C. Lighter traces signify increasing L concentration. The inset shows the changes in the apparent molar extinction coefficient at 429 nm (blue circles) and the fitting of the data to the corresponding temperature stage 1 binding model (grey line). See main text for details (b) Idem at 25 °C. (c) Idem at 5 °C, using temperature stage 2 binding model to fit the data. (d) (top) Values of the apparent binding constant for receptor **1Ch** for **L** for the addition of the first aliquot of the ligand at the temperatures tested (circles). The colours correspond to temperature stage 1 (red) and temperature stage 2 (blue). (bottom) Fraction of multivalent platform assembled, *x*MV, calculated as the ratio of all cluster forms of the receptor over total receptor (see eqn (12)) derived from the calculations at the temperatures tested in the absence of ligand (blue circles), and when the concentration of ligand is 2.5 mM (red circles). The lines have been added as visual aids.

The interaction of divalent ligand **L** with membrane anchored receptor **1Ch** was studied by means of UV-visible titration on vesicle samples with an in-membrane concentration *r***1Ch** = 0.01 over the temperature range under study. At this concentration the presence of the laterally assembled C form of **1Ch** is negligible in temperature stage 1 (above 20 °C). In this temperature stage, titration of **L** leads to changes in the UV-visible spectrum of **1Ch** that do not feature isosbestic points, consistent with the formation of complexes M**L** and M_2_**L** (Fig. 3 and Fig. 4a and b). The overall shape of the binding isotherms (Fig. 4a, b and ESI Fig. S3†) suggests that the binding affinity for the ligand increases as we approach 20 °C. Since *K*_m_ changes with the temperature are too small to be detectable, we attribute this observed change to an increase in the binding affinity of the ligand to the lipid bilayer that track changes in the membrane interface as seen in the UV-visible spectra of the receptor **1Ch** in the absence of ligand (Fig. 2c and d). This phenomenon is characterized by the membrane binding constant *K*_i_ (Fig. 3):2
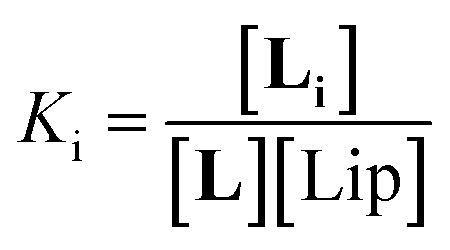


In eqn (2) [**Li**] is the concentration of membrane bound ligand, [**L**] that of ligand in solution and [Lip] that of lipid molecules, with all the concentrations referred to the total solution volume. The binding constant for the formation of M**L**, *K*_1_ (Fig. 3) can thus be written as a function of *K*_i_ as follows:3
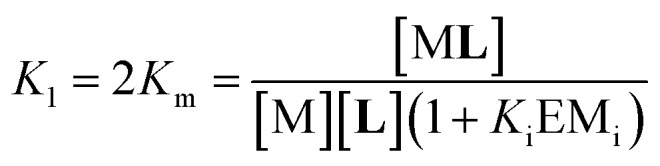
where EM_i_ is the effective molar concentration of the ligand in relation to the receptor in the hypothetical case of a membrane composed of pure monomeric receptor. The equilibrium constant for the formation of complex M_2_**L**, *K*_2_, can be written as (Fig. 3):4
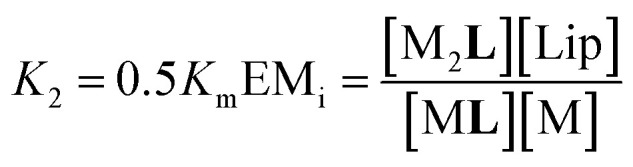


Eqn (2)–(4) (see ESI† for a detailed derivation), in combination with the mass balances, comprise a binding model that was used to fit the UV-visible titration data taken within temperature stage 1. *K*_m_ was determined from the experiments with **LR** and was entered as a fixed parameter. EM_i_ was determined from the titrations carried out at the highest temperature end (*i.e.* above 40 °C) where *K*_i_ is negligible. EM_i_ thus calculated has a value of 2.3 M, a reasonable value of effective concentration for molecular recognition events.^22^ For the fitting of the titration data below 40 °C, EM_i_ as well as *K*_m_ were entered as fixed parameters, with the *K*_i_ at each different temperature obtained as an adjustable parameter from the fitting procedure. The fitting to the model is excellent (Fig. 4a, b and ESI Fig. S3†). The values of *K*_i_ are small in all cases and decrease as the temperature increases (ESI Table S3†). At its highest (at 22 °C, the lowest temperature tested) *K*_i_ is only 1 M^−1^. This small affinity for the membrane has, however, a clear impact in increasing the extent of ligand binding to the receptor and the apparent binding affinity (Fig. 4d).

In temperature stage 2, below 20 °C, the monomeric and the laterally assembled cluster form of **1Ch** (M and C, respectively) co-exist. Their relative amounts at a given temperature depend on the corresponding in-membrane solubility, *r*_M,max_, or its reciprocal, the clustering constant *K*_c_. For the M form, the extent of ligand binding leading to M**L** depends on *K*_m_ as described in eqn (3). The clustered form of the receptor, C, has a larger affinity for **L** than the monomeric form, M, which is attributed to a combination of two factors. Firstly, the intrinsic increase in the binding affinity per binding site quantified by the modulation factor *M*_f_, which has been determined for the alkyl-imidazole binding sites of **L** using reference ligand **LR**. Thus, the binding constant for the formation of complex C**L**, *K*_4_, can be written as (Fig. 3):5
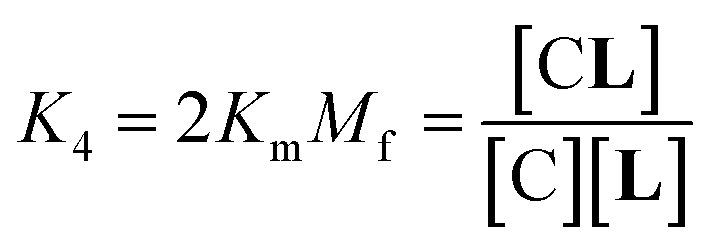


The second factor is the chelate effect at play within the domains or clusters C of receptor, quantified by the effective molarity, EM_c_. EM_c_ is the apparent concentration of the complementary binding sites for the formation of the complex C_2_**L**. The formation of this complex can therefore be written as:6
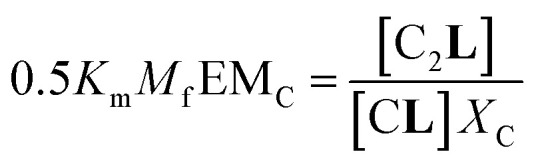
where *X*_C_ is the fraction of free binding sites within the clustered domains. It has to be noted that EM_C_ is different to the interface molarity EM_i_, as it applies to the apparent concentration of the binding sites within the clusters C, rather than a membrane that is composed of monomeric M. While eqn (5) and (6) illustrate the modulation of the binding brought about by the formation of the cluster C, it is possible to depict routes of formation of C_2_**L** that show how the converse is also true, that is, that the binding of the ligand modulates the lateral assembly into clusters. For example, the equilibrium regulated by constant *K*_5_ (eqn (7)) shows a mechanism by which the binding of L modulates the lateral assembly by recruitment of free monomers into the clustered domain (Fig. 3, eqn (6), see ESI Fig S4† for a summary of the different routes to formation of C_2_**L**):7
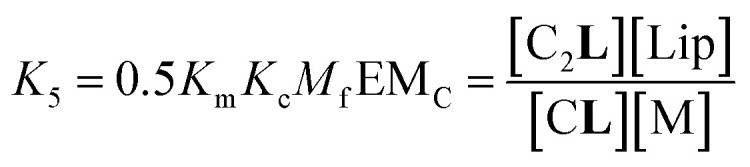


In temperature stage 2, M_2_**L** is one of the possible forms of the nucleus that lead to the growth of the clusters, as part of the nucleation-growth mechanism. The accumulation of this complex in this temperature stage can thus be considered negligible. It is noteworthy that when a large excess of **L** is added, the visible band assigned to C decreases (Fig. 4c). This result is consistent with a scenario in which a ligand-saturated membrane interface causes an increase of the in-membrane solubility of **1Ch**, leading to the de-assembly of clusters (Fig. 3 bottom). The ability of **Li** to stabilize the monomeric form of the receptor is likely rooted, at least in part, on the non-negligible amount of the protonated form of the imidazole moieties of the ligand.^23^ These cationic, protonated ligands will interact favourably with the anionic sulphonate moieties of **1Ch**. To incorporate this possibility to our model, we postulate that the membrane interface bound ligand, **Li**, binds to the cluster–ligand complex C**L,** detaching it from the cluster to yield the monomer–ligand complex form M**L**·**Li** (Fig. 3 bottom). The disassembly equilibrium constant of this process, *K*_da_, can be written as function of the relevant species as:8
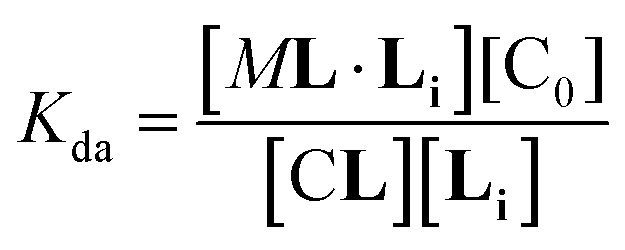
where [C_0_] is the concentration of all forms of cluster, that is the sum of [C], [C**L**] and 2× [C_2_**L**]. Together with the mass balances, eqn (2)–(5) and (7) and (8) comprise a clustering-binding model that describe the speciation at each temperature point of stage 2, *i.e.*, 5 to 20 °C (see ESI† for detailed derivation of the equations). In combination with the Lambert–Beer law, we use the model to fit the UV-visible data at each temperature point in this range. *K*_m_, EM_i_ and *M*_f_ had already been determined. EM_c_ has been estimated for **1Ch** and ligand **L** in our previous work and has a value of 1 M.^16^*K*_c_ at each temperature point was determined by the analysis of the clustering in the absence of ligand. The value of *K*_i_ was extrapolated from the data of *K*_i_ above 20 °C, using a van't Hoff approach (see ESI Fig S5 and S6, Table S4 and the associated discussion in the ESI† for details).^24^ These were entered as fixed values, leaving only the disassembly constant, *K*_da_, and the extinction coefficient of the different species to be optimized from the fitting of the UV-visible titration data. The resulting fit is excellent, which supports the accuracy of the model (Fig. 4c, ESI Fig. S7†). The value of *K*_da_ decreases as the temperature decreases (ESI Table S5†). Remarkably, *K*_da_ shows a linear correlation with the in-membrane solubility of the receptor, that is, the inverse of *K*_c_ (ESI Fig. S9†). As *K*_da_ can be visualised as the degree of solubility of the receptor–ligand complex in a ligand-saturated membrane, the correlation demonstrates that the solubility of the receptor–ligand complex in a ligand-saturated membrane is increased approximately 45 fold, in relation to the receptor in the membrane in absence of the ligand (see ESI Fig. S8 and S9, and associated discussion in the ESI†).

Our model implicitly assumes that, as far as ligand binding is concerned, the behaviour of the receptors C located at the boundary of the cluster or domain is indistinguishable to those located within the domain. The excellent fit of the data to the model is consistent with this assumption. Furthermore, the orientation of the receptors in the membrane also support to this view, as the porphyrin rings are known to sit approximately perpendicular to plane of the membrane.^25^ For receptors located at the edge there is thus a face that points toward the rest of the domain, where the bound ligand will be exposed to the same environment than those bound within the domain.

Overall, the models presented above allow us to determine speciation from which to derive the apparent binding constant, *K*_app_, at each temperature analysed at any concentration of **L**. The temperature trend of *K*_app_, calculated for the addition of the first aliquot of ligand, mirrors the observed changes in the UV-visible spectra and tracks the formation of the cluster C (Fig. 2c, d and 4d). These show a steep rise at around 20 °C and can be attributed to the multivalent effect at play in the cluster, C, enhanced by a favourable modulation factor, *M*_f_, for our ligand (*i.e.*, *M*_f_ > 1). Clearly, the presence of multiple molecules of receptor **1Ch** in close proximity within C render these domains multivalent platforms primed for the binding of multivalent ligands. We quantify the extent of formation of the multivalent platform, *x*MV, as the fraction of all forms of clustered receptor, [C_0_], over that of the total concentration of receptor, [**1Ch**]:9
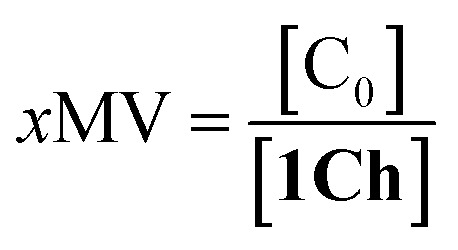


*x*MV was calculated from the titration data at the different temperature points analysed and the ligand concentrations used. There is a sharper increase of multivalent platform assembly in the presence of a moderate concentration of ligand (*i.e.* 2.5 mM) than in the absence of ligand (Fig. 4d). The point at 19.5 °C is especially noteworthy, where no platform is assembled (*i.e.* is switched OFF) unless the ligand is present (*i.e.*, it switches ON). These results point to the combination of two factors playing a role in the assembly of the multivalent platform: (i) the sudden increase in lipid–lipid interactions upon lipid phase transition which for DMPC on the vicinity of 19.5 °C (ref. 18) (ii) the binding of the ligand, which increases the stability of the clustered form and therefore results in an increase of the intensity of the ON switch at the point of phase transition when ligand is present.

Changes in equilibrium constants for self-assembly or phase transition have often been modelled using a van't Hoff approach. In the next section we apply this approach to our system. The aim is two-fold: on the one hand, to test whether the observed behaviour is indeed consistent with the phase change of the lipids. On the other hand, we aim to produce a global model that allows the prediction of the behaviour of the system as a response to multiple stimuli (*i.e.*, not just the concentration of all species involved but also the temperature) and that it is capable of recapitulating the modulation of the intensity of the experimentally observed ON signal.

### 2.3. Modelling the dependence of lateral assembly and ligand binding with the temperature

The van't Hoff equation is an approximation that assumes that the enthalpy associated to a given chemical reaction does not change with the temperature. This assumption requires that the heat capacity of reagents and products is the same. This is a reasonable assumption when we deal with molecular association processes that do not change the covalent framework of the species involved. In our system, the self-assembly behaviour of the receptor changes drastically around the melting temperature, *T*_m_, with some minor additional changes taking place at either side of *T*_m_. All these changes were modelled using the corresponding van't Hoff equation.

For the main change at *T*_m_ we used the van't Hoff equation adapted to lipid phase transition.^24^ We attribute the changes in clustering at *T*_m_ to the lower solubility of receptor **1Ch** in lipids in the bilayer when in the more rigid gel phase (G, below *T*_m_) compared to lipids in the lipid disordered phase (L_d_, above *T*_m_). The *G* state is characterized by the formation of large domains of ordered lipid molecules; the size of the domains depends on the cooperativity number, *n*_*C*_, which has been estimated at 200 for DMPC.^24^ It is therefore reasonable to assume that the molecules of the receptor, **1Ch**, are driven out of the ordered lipid domains and located preferentially between them, reducing the ability of the membrane to dissolve individual receptor molecules by a factor of *n*_*C*_. We define [Lip]_I_ as the apparent concentration of lipids available for the receptor molecules. Written as a function of the phase composition, [Lip]_I_ is:10
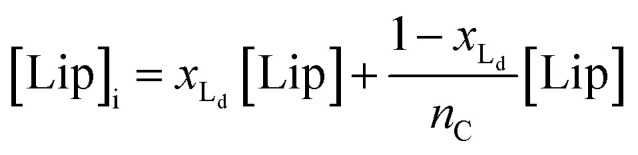
where *x*_L_d__ is the mol fraction of lipid in the L_d_ phase. The van'‘t Hoff equation for phase transition allows the calculation of *x*_Ld_ at any temperature and can be written as (see ESI† for the detailed derivation of eqn (11)):^24^11
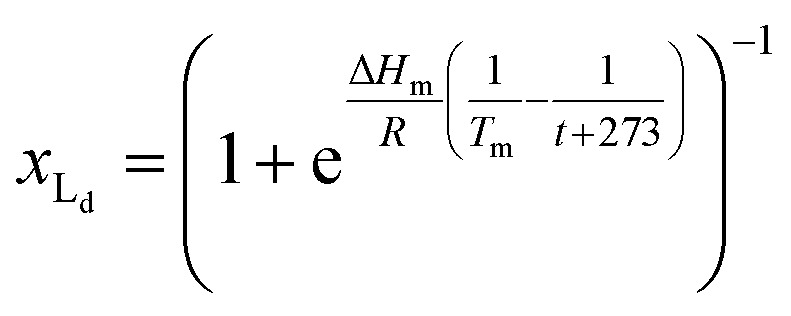
where Δ*H*_m_ is the enthalpy of the phase transition and *T*_m_ the melting temperature in Kelvin. Eqn (10) and (11) show that, when cooling below *T*_m_, there is a sudden drop in the amount of lipid molecules available to solvate the receptor. Consequently, the apparent in-membrane solubility drops dramatically. It follows that when the in-membrane concentration of **1Ch** is above this new, reduced solubility, there is a sudden jump in the lateral assembly of **1Ch** into clusters C (Fig. 2c and d).

Below *T*_m_ the solubility of **1Ch** further decreases with the temperature. This is shown by the increase of the clustering constant *K*_c_. We fit the increase of *K*_c_ with the temperature to the corresponding van't Hoff equation from which we obtained the apparent molar enthalpy and entropy for the in-membrane receptor clustering, Δ*H*_c_ and Δ*S*_c_ (ESI Fig S10,†Table 1).12
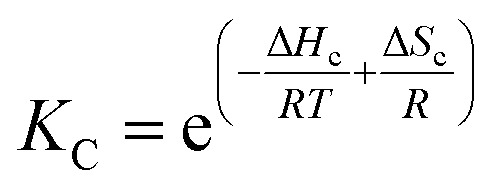


**Table 1:** Thermodynamic parameters associated with the lipid membrane transition and the clustering of the receptor **1Ch** in the membrane^a^

Δ*H*_m_^b^	*T*_m_	Δ*H*_C_	Δ*S*_C_
2930	292.6	−51.5	−133

a The values of molar enthalpy are reported in units of kJ mol^−1^, those of molar entropy in units of J mol^−1^ K^−1^ and the temperature in K.

b Value from ref. 24. The error, measured as twice the standard deviation and derived from the statistical analysis of the data fitting, is on the order of 25%.

The changes above *T*_m_ are attributed to changes in the lipid interface linked to the main phase transition, which give rise to both the solvatochromic shift observed and the increase in *K*_i_ (ESI Fig S5 and S6, Table S4†).

Eqn (10)–(12), together with eqn (1), modified to incorporate the apparent lipid concentration, [Lip]_i_, rather than the total lipid concentration, [Lip], and the mass balance constitute a model that account for the temperature dependent assembly of the receptor into clusters. Adding in the Lambert–Beer law, the model fits well to the experimental UV-visible data, allowing for small adjustments in the Lambert–Beer law equations to account for the solvatochromic shift experienced by the monomeric form of the receptor M as we approach *T*_m_ (see ESI† for details of the model). When fitting the data, the thermodynamic parameters for the lipid phase change and receptor clustering (Table 1) were entered as fixed values, as was the cooperativity number *n*_C_ (see ESI† for details). Therefore, the only optimized parameters were *T*_m_ and the extinction coefficient of the different species. The fit of the model to the UV-visible data is excellent (ESI Fig S11†). The value of *T*_m_ obtained from the fitting is 292.6 K (19.6 °C), which is consistent with literature data for vesicles composed of DMPC.^18^

For the binding of the ligand, we assume that *K*_m_ is independent of the temperature, based on the lack of a discernible variation of *K*_m_ for the reference ligand **LR** (ESI Table S2†). The binding of the ligand to the membrane is described by eqn (2), while the changes in *K*_i_ are described by eqn (S76) (see ESI†). The formation of complexes M**L** and C**L** are described in eqn (3) and (5). The lateral assembly of **1Ch** into C and the formation of M_2_**L** and C_2_**L** is described by eqn (1), (4) and (7). These equations are modified, replacing the concentration of lipid [Lip] by the apparent concentration [Lip]_i_, whose temperature dependence is recapitulated by eqn (10) and (11). Changes in *K*_c_ with the temperature are described by eqn (12). Together with the corresponding mass balances, these equations comprise a global assembly-ligand binding model that accounts for the speciation and UV-visible spectra observed at any system composition and any temperature (see full model details in the ESI†).

**Fig. 5:**
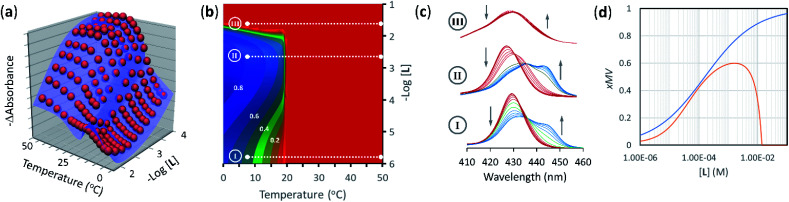
(a) Changes in absorbance at 429 nm of membrane anchored **1Ch** in DMPC vesicles upon changes in the temperature and the concentration of ligand (red spheres), fit to a global clustering-binding model (blue surface). The in-membrane concentration of **1Ch** was *r***1Ch** = 0.01. (b) Simulated changes in the formation of receptor multivalent platforms (*x*MV, see eqn. (9)) as a function of the temperature and the concentration of ligand **L**. Each change of the colour tonality represents an increase of 0.1 in *x*MV. The dotted white lines labelled I, II and III correspond to the experimental conditions that give rise to the corresponding spectra displayed in panel (c). (c) Changes in the Soret band region of the UV-visible spectra of membrane anchored **1Ch** upon changes in the temperature. The grey arrows indicate the direction of change as the temperature is decreased. The concentration of ligand was 0 mM (bottom spectra, I), 2 mM (middle spectra, II) and 20 mM (top spectra, III). *r***1Ch** was 0.01 in all cases. (d). Intensity of the ON signal, measured as the fraction of multivalent platform, *x*MV, upon switching ON (*i.e.*, cooling to 19.5 °C, just below the *T*_m_) and as a function of the concentration of ligand. The red trace was calculated from speciation derived from the global model. The blue trace was calculated using eqn (14) (*r***1Ch** = 0.01).

The model was fit to the UV-visible data at all temperatures and ligand concentrations. All the thermodynamic parameters have already been determined and were entered as fixed parameters, with only the colour of the species (*i.e.*, the corresponding extinction coefficients) optimized. The fitting of the global model to the data is excellent, tracking closely all the observed changes, both major and minor (Fig. 5a). Major changes include the sudden increase in UV-visible signal around the *T*_m_. This large change in the UV-visible signal reflects the sudden lateral assembly at *T*_m_, that becomes sharper as the concentration of ligand increases. At very large concentrations of the ligand, the change in the UV-visible signal around *T*_m_ becomes very small, reflecting the fact that large excess of ligand inhibits rather than promotes the lateral assembly of the receptor into multivalent clusters or domains.

The changes in receptor distribution in the membrane are clearly shown when the model is used to simulate the extent of assembly of the multivalent platform, *x*MV, as the temperature and the ligand concentration are changed (Fig. 5b and c).

Our mathematical model is uncomplicated in that it is built on straightforward algebraic relationships derived from the chemical equilibria involved and the van't Hoff equation to predict the system composition and properties. Although implementation of the global model requires specialist software, the ligand modulation of a bimodal assembly switch can be recapitulated into a simple mathematical expression. For our receptor, we measure the intensity of the ON response as the fraction of receptor that assembles into multivalent platforms *x*MV (eqn (9)). In the absence of ligand, upon triggering the switch (*i.e.* cooling below *T*_m_), the value of *x*MV depends only on the excess of in-membrane concentration of the receptor in relation to the in-membrane solubility. Since the binding of the ligand modulates the in-membrane solubility, *x*MV can be written as function of the ligand concentration as follows (see ESI† for detailed derivation):13
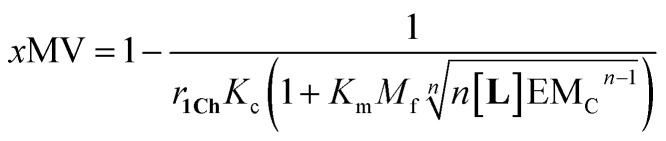
where *n* is the number of binding sites on the ligand (2 for **L**). When the in membrane concentration of receptor equals the solubility just below the *T*_m_, the deployment of receptor domains (that is, the growth of *x*MV) does not take place in an ON/OFF bimodal fashion, but rather increases steadily as the solubility decreases with the temperature. In our experiments, this scenario is observed for *r***1Ch** = 0.01 in the absence of ligand (Fig. 2c). At this temperature point, eqn (13) can be simplified to:14
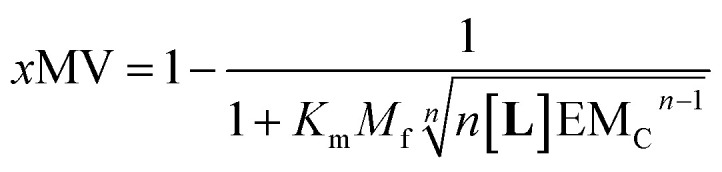


Eqn (14) shows that, in these conditions, the intensity of the ON signal depends on the pairwise binding parameters (*i.e.* the intrinsic binding affinity), the number of binding sites and the concentration of ligand. Crucially, in these conditions, the ON/OFF deployment of multivalent receptor platforms requires the presence of the ligand. In our experiments, this scenario is observed for samples with *r***1Ch** = 0.01 in the presence of a concentration of ligand in the millimolar region (Fig. 5b, c and d). From eqn (14) we can derive an expression that allows us to determine the minimum concentration of ligand required to obtain the minimum ON signal intensity desired. For example, for the deployment of 50% of the multivalent platform we have that:

15 [**L**] = *n*^−1^(K_m_M_f_)^−*n*^EM_C_^*n*−1^

Eqn (15) clearly shows that the concentration of ligand required to initiate the deployment of the multivalent platform decreases with the number of binding sites, the value of the intrinsic binding affinity (*K*_m_), and that of the cooperativity parameters *M*_f_ and EM_C_.

Eqn (13) and (14) are simplifications that nonetheless predict the approximate behaviour of membrane anchored receptors in response to the presence of their ligands. In particular, they show that ligands with small binding affinities can greatly enhance a temperature dependent ON/OFF membrane-receptor multivalent switch, provided that the concentration of the ligand is large enough, or that the ligand is heavily multivalent (*i.e.*, *n* is large). Such is the case in our system, where an ON/OFF switch is achieved with intrinsic binding constants below 100 M^−1^. The behaviour of our system also shows the limitations of using a simple equation to describe the system. According to eqn (13), the level of response is larger as the concentration of ligand increases. In reality, at large ligand concentration we see disassembly of the multivalent platforms, due to non-specific interaction of the ligand with both the membrane and the receptor (Fig. 5d). This observation highlights the fact that non-specific interactions are likely to interfere with the molecular switch in those cases where low binding affinity requires the use of large concentration of the ligand.

## 3. Conclusions

In summary, in this work we have used spectroscopic data of a well-defined, chemically simple lipid membrane system to derive a clustering-binding model and describe its behaviour as a function of the system composition (*i.e.* the relative concentration of ligand, lipid and receptor) and the temperature. To the best of our knowledge, the development of such a global model based on discrete pairwise binding parameters is unprecedented. Our model clearly shows that the behaviour of the membrane receptors is largely dictated by the properties of the lipid membrane, but that their behaviour can be finely tuned *via* the self-assembly of the receptor and the presence of a ligand in solution. The model accurately describes the bimodal ON/OFF assembly of the receptor into multivalent platforms, triggered by a temperature switch. The modulation of the ON signal, from nil to full response, is induced by the action of a small ligand in solution. The model also highlights the limitations of weakly binding ligands as modulators of the ON signal, due to the fact that the large concentration required for full deployment leads to additional interactions that may interfere with the function of the switch. The modulation of the signal can be recapitulated into a simple equation (eqn (14)) that offers a good approximation of the system response to the ligand. Current research efforts in our laboratory are focused on developing membrane adhesion and small molecule release switches based on the triggering of the multivalent platforms described here. This work brings their development closer and will open the door to the design of protocells programmed to adhere to other protocells or to living cells upon stimulus, or to allow a fine control of the release of their contents. The former can be applied to the development of hierarchically assembled proto-tissues.^12,13^ The latter, to develop stimuli-responsive drug delivery vehicles. Our work thus complements the recent development of signal amplification switches in lipid vesicles.^26–28^ Finally, it is worth noting that the behaviour of our system is reminiscent to that of biological membrane receptors in response to messengers.^29^ Clearly, many of these biological systems are qualitatively and quantitatively different to the one described here. For example, the well know tyrosine kinase relies on dimerization of a receptor, rather than on the formation of extended domains, with very strong binding affinities.^30^ Our mathematical model may nonetheless find application in the study of cell adhesion phenomena that rely on assembly of many copies of relatively weakly binding ligands and receptors.^31,32^

## Author contributions

AG performed the initial experiments and analysed the data. BW performed additional temperature-variable experimnets, analysed the data and co-worte the manuscript, MP-S performed monovalent ligand control experiments, analysed the data and co-wrote the manuscript. LM analysed the data and co-worte the manuscript, ST designed the research, analysed the data and wrote the manuscript.

## Conflicts of interest

There are no conflicts to declare.

## Supplementary Material

SC-012-D1SC01598B-s001
