# Supplementary material for: How the biomimetic assembly of membrane receptors into multivalent domains is regulated by a small ligand
Source: Chem Sci. 2021 Apr 30;12(22):7800–8. doi: 10.1039/d1sc01598b (PMC8188472; doi:10.1039/d1sc01598b)
Supplement: SC-012-D1SC01598B-s001 [file SC-012-D1SC01598B-s001.pdf]

Electronic Supplementary Material (ESI) for Chemical Science.
This journal is © The Royal Society of Chemistry 2021

How the biomimetic assembly of membrane receptors into multivalent domains is regulated by a small ligand

Anna Grochmal, Ben Woods, Lilia Milanesi, Manuel Perez-Soto, and Salvador Tomas*

Supplementary Information

Methods

General. Solvents, chemicals and ligand $\mathbf{L_R}$ were obtained from commercial sources and used without further purification. Receptor **1Ch** and ligand **L** were synthesised and purified as described elsewhere.[7,16] UV-Visible absorbance spectra were recorded with a Varian Cary 3 equipped with a Peltier- temperature controlled sample holder. All the experiments were carried out in sodium carbonate buffer at pH 10.3. The buffer was prepared by dissolving $NaHCO_3$ up to 100 mM in de-ionized water and adjusted to pH 10.3 by adding the appropriate amount of a solution of NaOH 5M.

Liposome preparation. A stock solution of lipids was prepared by dissolving synthetic dimyristoylphosphatidylcholine (DMPC) in ethanol, with a concentration of 20 mmol/L. A stock solution of **1Ch** in ethanol with a concentration 1 mM was also prepared. To prepare the liposomes, DMPC and **1Ch** stock solutions were mixed in the appropriate proportions. The solvent was removed under reduced pressure, using a rotary evaporator set at 40 ^{o}C for a minimum of 30 minutes. The solid was then re-suspended in the buffer by vortexing for 2 minutes. Excess air and foam in the suspension were removed by sonicating the vortexed sample for 20 seconds in an ultrasound bath. The suspension was then extruded (Avanti Mini-Extruder) through 200 nm pore-size polycarbonate filters a minimum of 31 times. The resulting vesicle suspension was used within 6 hours of preparation to minimize distortions due to the presence of lyso-phospholipids from hydrolysis of the DMPC.

UV-visible spectra. The spectra of all the samples between 360 and 540 nm was recorded. In all the cases, the absorbance of the Soret band is located between 385 and 465 nm and outside of this range **1Ch** does not absorb. Therefore, the data between 360-385 nm and 470-540 nm was used to re-construct the baseline due to the light scattering of the liposomes, by fitting a third polynomial function which was then subtracted from the relevant spectra. The corrected spectra were used to calculate the binding parameters and to build up the figures.

Data fitting. We used Micromath Scientist 3.0 to fit the experimental UV-visible data to the appropriate model, which were entered as a system of non-explicit equations. The absorbance data

of up 4 different wavelengths (i.e. those wavelengths where the changes are the largest and clearest) were entered as dependent variables. Most of the binding parameters were previously known and therefore entered as fixed constants. The extinction coefficient parameters were constrained between values that were consistent with typical values for the Soret band of Zn metalloporphyrin moieties (i.e. from 0 to 400,000 $M^{-1}cm^{-1}$). To build the display figures that show a surface fitting (Supplementary Fig. 10 and Fig. 5a) we used the program PovRay v3.7. The experimental values for the extinction coefficient were entered as the coordinate of the centre of the spheres, and the fitted surface was generated using the optimized parameters.

Calculating apparent binding constant *K_{app}* (Figure 4d). The apparent binding constant was calculated using the equation below:

$$K_C = \frac{[1Ch \cdot L]}{[1Ch][L]} \qquad \text{Eq. S1}$$

Where **1Ch·L** is the sum of concentrations of all the forms of receptor bound to ligand:

$$[1Ch \cdot L] = [ML] + 2[M_2L] + [CL] + 2[C_2L] \qquad \text{Eq. S2}$$

And [**1Ch**] represents all forms of the receptor that are ligand free:

$$[1Ch] = [M] + [C] \qquad \text{Eq. S3}$$

The concentration of each of the species was calculated using the relevant model at the corresponding temperature stage, using the previously known and optimized binding parameters (Fig. 4d).

Calculating *xMV* . The concentration of relevant species (Eq. 12) was calculated using the relevant model at the corresponding temperature stage, inserting both previously known and the optimized binding parameters. For figure 4d, the concentration of each of the species was calculated using the relevant model at the corresponding temperature stage. For figure 5b the global model was used to calculate the concentration of species to build up the simulation.

Model for the in-membrane nucleation-growth assembly of the receptor

For the isodesmic assembly model two forms of the receptor, monomer *M* and the clustered form *C*, are considered to be dissolved in their respective solvents, either in lipids (*Lip*) or the receptor itself (**1Ch**). The partition process can be written as:

$$M\ (Lip) \rightleftharpoons C\ (\mathbf{1Ch}) \qquad \text{Eq. S4}$$

for which a clustering constant K_c can be written, as a function of the bulk volume concentrations:

$$K_C = \frac{[C][Lip]}{[M][1Ch]} \qquad \text{Eq. S5}$$

This equilibrium can also be thought of as a binding event in which a molecule of the monomeric form of the receptor *M,* initially bound to a lipid molecule *Lip*, associates with another molecule of the receptor (**1Ch**). This would yield a molecule of the clustered form of the receptor, *C,* and release the lipid molecule *Lip* that was associated to *M*. That is, a molecular exchange that can be written as:

$$M + \mathbf{1Ch} \rightleftharpoons C + Lip \qquad \text{Eq. S6}$$

For nucleation growth the cluster will only grow if *M* binds with **1Ch** molecules that are either part of a nucleus (composed of at least two molecules of **1Ch**) or as part of the growing cluster, but not as monomer *M*. We can therefore write the nucleation growth process as:

$$M + (C \text{ or } Nuc) \rightleftharpoons C + Lip \qquad \text{Eq. S7}$$

Where *Nuc* is the nucleus. Thus, the clustering constant that defines the extent of clustering can be written as a function of the bulk volume concentrations of species:

$$K_C = \frac{[C][Lip]}{[M]([C] + [Nuc])} \qquad \text{Eq. S8}$$

For simplicity of notation we define the species C_0, as the sum of all clusters and nucleus, that is:

$$[C_0] = [C] + [Nuc] \qquad \text{Eq. S9}$$

Which substituted in Eq. 6 is:

$$K_C = \frac{[C][Lip]}{[M][C_0]} \qquad \text{Eq. S10}$$

For a highly cooperative assembly, under conditions where *C* forms, the concentration of the nucleus is much lower than that of *C* or *M*. We can therefore assume that:

$$[C_0] \approx [C] \qquad \text{Eq. S11}$$

which combined with Eq. 4 gives:

$$K_C = \frac{[Lip]}{[M]_{max}} \qquad \text{Eq. S12}$$

Therefore, the maximum in-membrane concentration of the monomer (i.e. the in-membrane solubility) relates to the clustering constant as follows:

$$r_{M,max} = \frac{1}{K_C} \qquad \text{Eq. S13}$$

Calculating the clustering constant K_c at specific temperatures during temperature stage 2 (t_i < 20 °C)

In the growth regime we can assume that there are two different species of **1Ch** in the membrane, the monomer *M* and the clustered form *C*. The absorbance observed at any given wavelength, A_{obs}, can be written as:

$$A_{obs} = \varepsilon_C[C] + \varepsilon_M[M] \qquad \text{Eq. S14}$$

Where ε_C and ε_M are the extinction coefficients of the pure *C* and *M* forms of the receptor at the wavelength being monitored. The apparent extinction coefficient, ε_{app}, can thus be written as:

$$\varepsilon_{app} = \varepsilon_C\frac{[C]}{[1Ch]} + \varepsilon_M\frac{[M]}{[1Ch]} \qquad \text{Eq. S15}$$

Which combined with equation S5 yields:

$$\varepsilon_{app} = \varepsilon_C\frac{[C]}{[Lip]r_{1Ch}} + \varepsilon_M\frac{[M]}{[Lip]r_{1Ch}} \qquad \text{Eq. S16}$$

Which can be re-arranged as:

$$\varepsilon_{app} = \varepsilon_C(1 - \frac{[M]}{[Lip]r_{1Ch}}) + \varepsilon_M\frac{[M]}{[Lip]r_{1Ch}} \qquad \text{Eq. S17}$$

In the growth regime, and substituting in equation 3, equation S15 can be re-written as:

$$\varepsilon_{app} = \varepsilon_C(1 - \frac{1}{K_C r_{1Ch}}) + \frac{\varepsilon_M}{K_C r_{1Ch}}$$ Eq. S18

Which can be re-arranged as:

$$\varepsilon_{app} = \varepsilon_C - \frac{\varepsilon_C}{K_C r_{1Ch}} + \frac{\varepsilon_M}{K_C r_{1Ch}}$$ Eq. S19

Therefore, the increase in apparent extinction coefficient, $\Delta\varepsilon_{app}$, can be written as:

$$\Delta\varepsilon_{app} = \Delta\varepsilon(1 - \frac{1}{K_C r_{1Ch}})$$ Eq. S20

$\Delta\varepsilon$ is common to the data at all temperatures. We can thus write for any temperature (t_i) below 20 °C:

$$\Delta\varepsilon_{app} = \Delta\varepsilon(1 - \frac{1}{K_{C,ti} r_{1Ch}})$$ Eq. S21

where $K_{C,ti}$ is the clustering constant at temperature t_i. Equation S21 was used to fit the experimental data of $\Delta\varepsilon_{app}$ at different temperatures and values of $r_{\mathbf{1Ch}}$ simultaneously (Supplementary Fig. S1). The K_C obtained from the fitting are shown in Supplementary Table S1, along with the corresponding maximum in-membrane concentration of *M*, $r_{M,max}$ (that is, the in-membrane solubility of **1Ch**), calculated as the inverse of K_C.

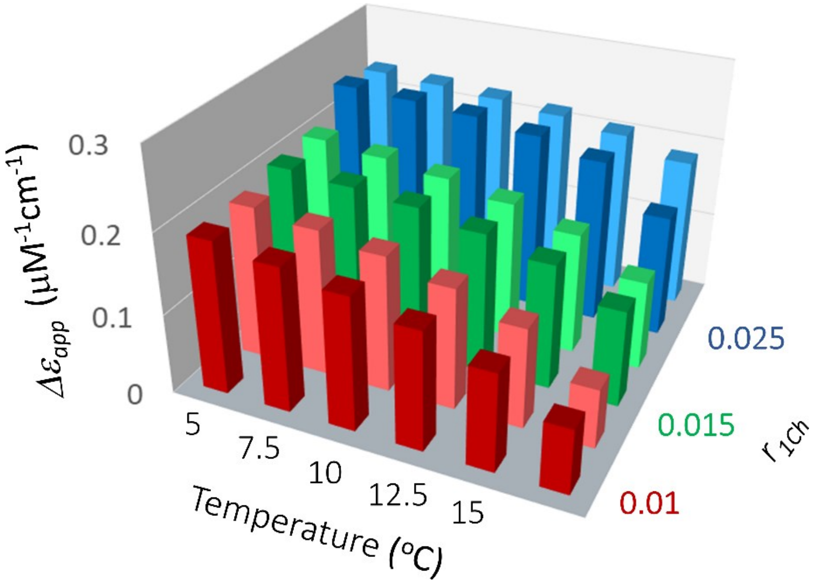

Supplementary Fig. S1. Graphical representation of the values of $\Delta\varepsilon_{app}$ at 445 nm for samples with different in-membrane concentrations, $r_{\mathbf{1Ch}}$, and at different temperatures. Each colour applies to samples at different values of $r_{\mathbf{1Ch}}$. The darker columns represent the experimental values of $\Delta\varepsilon_{app}$ and

the lighter values obtained from the best the fit to equation. S21. See Supplementary table 1 for numerical values of K_c and $r_{M,max}$.

Supplementary Table S1. Values of the K_C and $r_{M,max}$ at different temperatures in the stage 2 (i.e. below 20 °C) obtained from the fitting of the UV-Visible data. The error of the measures, quantified as twice the standard deviation, is in the order of 25%.

t (°C)	5	7.5	10	12.5	15	17.5
K_C	370	330	280	235	190	140
$r_{M,max}$	0.0027	0.0030	0.0035	0.0042	0.0052	0.0072

Ligand binding

Reference ligand $\mathbf{L_R}$

Methyl imidazole, $\mathbf{L_R}$, binds to the membrane anchored receptor **1Ch** to form the complex **1Ch•L**$_\mathbf{R}$, with an apparent constant K_{app}:

$$K_{app} = \frac{[1Ch \cdot L_R]}{[1Ch][L_R]}$$ Eq. S22

The mass balances are:

$$[1Ch]_0 = [1Ch] + [1Ch \cdot L_R]$$ Eq. S23

$$[L_R]_0 = [1Ch \cdot L_R] + [L_R]$$ Eq. S24

According to the Lambert-Beer law, at any given wavelength of the Soret band the apparent extinction coefficient is the ratio between the observed absorbance, A_{obs}, at this wavelength and the total concentration of receptor:

$$\varepsilon_{app} = \frac{A_{obs}}{[1Ch]_0}$$ Eq. S25

which can be written as a function of the concentration of the chromophore species as:

$$\varepsilon_{app} = \varepsilon_{1Ch}\frac{[1Ch]}{[1Ch]_0} + \varepsilon_{Ch \cdot L_R}\frac{[1Ch \cdot L_R]}{[1Ch]_0}$$ Eq. S26

K_{app} was determined from fitting the UV-visible data at the wavelength corresponding to the largest change in absorbance (429 nm) to the model composed of the system of equations S22-24 and S26 (see Methods for details on the experiments and the software). (Supplementary Fig. S2, Supplementary Table S2).

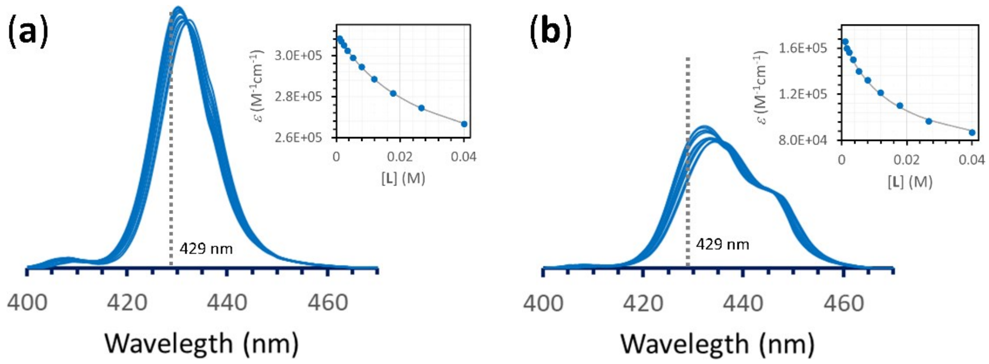

Supplementary Fig. S2 (a) Changes in the Soret band region of the UV-visible spectrum of membrane embedded **1Ch** upon addition of increasing amounts of reference ligand $\mathbf{L_R}$ at 40°C. The inset shows the changes in the apparent molar extinction coefficient at 429 nm (blue circles) and the fitting of the data to a 1 to 1 binding model (grey line) from which a value of apparent binding constant, K_{app}, of 38 M^{-1} was derived (see Supplementary Table 2). (b) Idem at 5°C. The inset shows the changes at 429 nm. The value of apparent binding constant, K_{app}, derived was 70 M^{-1}.

Supplementary Table S2. K_{app} for ligand $\mathbf{L_R}$ at different temperatures, calculated from the fitting of UV-visible titration data at 429 nm to the 1 to 1 binding model. The error quoted is twice the standard deviation of 3 independent measures.

t (°C)	5	30	40	50
K_{app} (M^{-1})	70 ± 8	39 ± 5	37 ± 4	38 ± 5

In temperature stage 1 (t > 20 °C) the only form of receptor present is monomer M, which can form the complex $M\mathbf{L_R}$. In this temperature range, K_{app} equals the microscopic constant K_m, and can be written as:

$$K_m = \frac{[ML_R]}{[M][L_R]} \quad \text{Eq. S27}$$

The value of K_m, calculated as the average of the values above 30 °C , is 38 M^{-1}.

In stage 2 (t < 20 °C) K_{app} can be written as:

$$K_{app} = \frac{[CL_R] + [ML_R]}{([C] + [M])[L_R]} \qquad \text{Eq. S28}$$

The binding affinity for the clustered form of the receptor can be written as a function of the binding affinity for the monomer, K_m, and the modulation factor M_f, which quantifies the change in binding affinity of the receptor for the ligand upon clustering:

$$K_m M_f = \frac{[CL_R]}{[C][L_R]} \qquad \text{Eq. S29}$$

Combining equations S27- S29 results in:

$$K_{app} = K_m \frac{[M]}{[1Ch]} + K_m M_f \frac{[C]}{[1Ch]} \qquad \text{Eq. S30}$$

Which can be written as a function of the in-membrane concentration of the receptor, r_{1Ch}, and clustering constant, K_C, as:

$$K_{app} = \frac{K_m}{K_C r_{1Ch}} + K_m M_f (1 - \frac{1}{K_C r_{1Ch}}) \qquad \text{Eq. S31}$$

After rearranging we show that M_f can be written as a function of the different binding constants:

$$M_f = \frac{K_{app} r_{1Ch} K_C - K_m}{K_m r_{1Ch} K_C - K_m} \qquad \text{Eq. S32}$$

Substituting the appropriate values of K_m and r_{1Ch} (i.e. 38 M^{-1} and 0.01) and those of K_{app} and K_C (from Supplementary Table 1 and 2 at 5°C), gives:

$$M_f = 2.2 \qquad \text{Eq. S33}$$

Derivation of the equations for the binding of divalent ligand L.

Temperature stage 1 (t > 20 °C)

The experiments with monovalent ligand $\mathbf{L_R}$ have allowed the determination of the binding constant (K_m) and the modulation factor, M_f, for a ligand that bears a chemically identical binding site to those in divalent ligand **L**. It is therefore assumed that the binding constant of **L** for the receptor is the same, if it is statistically corrected to account for the presence of two binding sites in **L**, while each of the imidazole moieties should have the same M_f for the binding to the receptor. According to the

UV-visible data, in temperature stage 1 (i.e. above 20 °C) the self-assembly of **1Ch** is negligible for samples with r_0 = 0.01 (Fig 2b), and the receptor is therefore found in the monomeric form *M.* In this temperature stage, the binding of divalent **L** can lead to the formation of a 1 to 1 complex, *M***L**, and by binding to the second binding site of the ligand, the 2 to 1 complex, M_2**L**, is formed (Fig 3). The formation of the complex *M***L** depends on K_m according to equation 4:

$$K_1 = 2K_m = \frac{[ML]}{[M][L]} \qquad \text{Eq.S34}$$

Where 2 is the statistical factor that accounts for the fact that **L** bears 2 identical binding sites, compared to one binding site for $\mathbf{L_R}$. Eq. S34 is applicable for the binding of the ligand found in solution. The ligand can also bind to the membrane interface, according to Eq. 2:

$$K_i = \frac{[L_i]}{[L][Lip]} \qquad \text{Eq. 2}$$

From the point of view of membrane bound *M*, the membrane-bound ligand $\mathbf{L}_i$ has an apparent concentration, $[\mathbf{L}]_{app}$, that is different to the concentration in relation to the bulk solvent, $[\mathbf{L}_i]$. This apparent concentration can be written as:

$$[L]_{app} = [L_i]\frac{EM_i}{[Lip]} \qquad \text{Eq. S35}$$

For membrane bound ligand binding to the receptor:

$$K_1 = 2K_m = \frac{[ML]}{[M][L]_{app}} \qquad \text{Eq. S36}$$

Substituting in Eq. S35 to Eq. S36 gives:

$$K_1 = 2K_m = \frac{[ML][Lip]}{[M][L_i]EM_i} \qquad \text{Eq. S37}$$

Thus, for the case where [*Lip*] = [*M*] we have that:

$$K_1EM_i = 2K_mEM_i = \frac{[ML]}{[L_i]} \qquad \text{Eq. S38}$$

From Eq. S38 it can be seen that EM_i is the effective molarity of *M* in the interface for the formation of complex *M***L**, in the hypothetical situation in which the concentration of lipid equals that of monomeric receptor *M* (i.e. a membrane composed of pure receptor in the form *M*).

In our system, both the $\mathbf{L}_i$ and **L** forms of the ligand are present at the temperatures studied. Eq. 34 can thus be written as

$$K_1 = 2K_m = \frac{[ML]}{[M]([L] + [L]_{app})} \qquad \text{Eq. S39}$$

Combining Eq. S39 with Eq. S35 we have that:

$$K_1 = 2K_m = \frac{[ML]}{[M]([L] + [L_i]\frac{EM_i}{[Lip]})} \qquad \text{Eq. S40}$$

And substituting in Eq. 2 we have that:

$$K_1 = 2K_m = \frac{[ML]}{[M]([L] + [L]K_iEM_i)} \qquad \text{Eq. S41}$$

Which can be re-arranged to:

$$K_1 = 2K_m = \frac{[ML]}{[M][L](1 + K_iEM_i)} \qquad \text{Eq. 3}$$

The formation of complex M_2**L** from M**L** and M that are located in the same membrane depends on the in-membrane concentrations of these species. The binding constant for this process, K_2, can thus be written as:

$$K_2 = \frac{r_{M2L}}{r_{ML}r_M} \qquad \text{Eq. S42}$$

r_{M2L}, r_{ML} and r_M are calculated as the ratio of the concentration, in relation of the total solution volume, of the corresponding species over that of the lipid. Eq. S42 can thus be written as a function of K_m and the concentration of the species in relation to the total volume of solvent as (Fig 3):

$$K_2 = 0.5K_mEM_i = \frac{[M_2L][Lip]}{[ML][M]} \qquad \text{Eq. 4}$$

Where the factor 0.5 is the statistical correction factor that accounts for the fact that dissociation of the complex by either of the two **L** binding sites re-generates the binding partners (M and M**L**).

Model for temperature stage 1

Equilibrium equations

$$K_i = \frac{[L_i]}{[L][Lip]}$$ Eq. 2

$$2K_m = \frac{[ML]}{[M][L](1 + K_i EM_i)}$$ Eq. S43

$$0.5K_m EM_i = \frac{[M_2L][Lip]}{[M][ML]}$$ Eq. S44

Mass balances:

$$[1Ch] = [M] + [ML] + 2[M_2L]$$ Eq. S45

$$[L]_0 = [L_i] + [L]$$ Eq. S46

$$\varepsilon_{app} = \frac{A_{obs}}{[1Ch]}$$ Eq. S47

$$\varepsilon_{app} = \frac{1}{[1Ch]}\left(\varepsilon_M[M] + \varepsilon_{ML}[ML] + \varepsilon_{M2L}[M_2L]\right)$$ Eq. S48

The concentrations of *M***L** and M_2**L** are negligible in relation to those of $\mathbf{L}_i$ and **L**, and have therefore been disregarded for the mass balance of the ligand (Eq. S46). The apparent extinction coefficient, ε_{app}, at 4 different wavelengths was fitted to this model (Fig. 4a and 4b, Supplementary Fig. S3, Supplementary Table S3). See methods for details on the fitting procedure. The value of EM_i, obtained from the fitting of the data at the highest temperature point, is 2.3 ± 0.5 M.

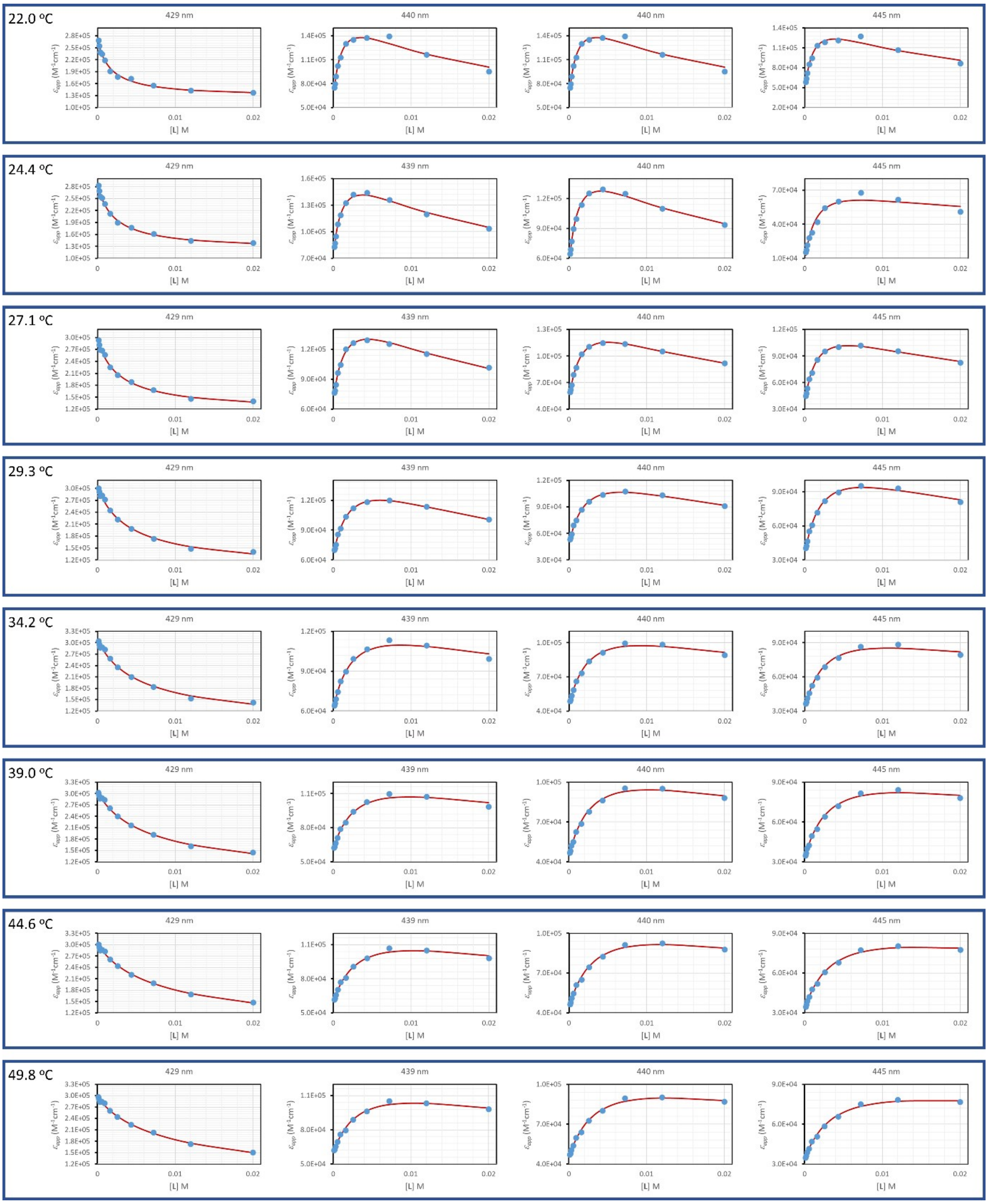

Supplementary Fig. S3. Values of ε_{app} for samples of **1Ch** in DMPC vesicles ($r_{\mathbf{1Ch}}$ = 0.01, [**1Ch**] = $2\text{x}10^{-6}$ M) at increasing concentrations of ligand **L** (solid circles) and the fitting to the stage 1 model (solid red line).

Supplementary Table S3. Values of K_i obtained from the fitting of the absorbance data at 4 fixed wavelengths in temperature stage 1.

t (°C)	22.0	24.4	27.1	29.3	34.2	39.0	44.6	49.8
K_i (M^{-1})	1.0	0.78	0.53	0.24	0.10	<0.1	<0.1	<0.1

The error in the measure is of the order of 20 %.

Temperature stage 2 (t < 20 °C)

In temperature stage 2, below 20°C, there are two dominant forms of the receptor, the monomer *M* and the cluster *C*, whose relative amounts depend on the in-membrane solubility, $r_{M,max}$, or its reciprocal, the clustering constant K_C. The clustering constant can be written as:

$$K_C = \frac{[C][Lip]}{[M][C_0]} \qquad \text{Eq. S10}$$

In the absence of ligand and for a strongly cooperative lateral self-assembly, we can assume that [C_0] is approximately equal to [*C*]. In the presence of ligand, C_0 is the sum of ligand bound and free forms of the clustered form of the receptor, that is:

$$[C_0] = [C] + [CL] + 2[C_2L] \qquad \text{Eq. S49}$$

The fraction of free binding sites within the cluster, xC, is defined as:

$$x_C = \frac{[C]}{[C_0]} \qquad \text{Eq. S50}$$

L binds to the cluster *C* to form the complex *C***L** according to the equilibrium equation 5 (Fig 3)

$$K_4 = 2K_m M_f = \frac{[CL]}{[C][L]} \qquad \text{Eq.5}$$

Where the modulation factor M_f accounts for the increase in the binding affinity of **L** for *C* in relation to *M*. The formation of complex C_2**L** can take place via several routes. One route is via the binding of the second free binding site of the ligand within *C***L** with nearby free *C*. The chelate effect at play for this binding event is quantified by the effective molarity of the cluster, EM_C. The binding constant can be written as a function of the concentration of the species involved as:

$$0.5K_m M_f EM_c = \frac{[C_2L]}{[CL]X_C} \qquad \text{Eq. 6}$$

Other routes to the formation of C_2**L** include that of binding of monomeric receptor M to complex *C***L**:

$$K_5 = 0.5K_m K_c M_f EM_c = \frac{[C_2L][Lip]}{[CL][M]} \qquad \text{Eq. 7}$$

The route relating to Eq. 7 is shown in Fig. 3. Alternative routes are shown in Supplementary Fig. S4. These routes can be written as a combination of the five independent equilibria shown in Fig. 3. Thus, considering the relevant equilibrium shown for the formation of C_2**L**:

$$K_1 = 2K_m = \frac{[ML]}{[M][L]} \qquad \text{Eq. S51}$$

$$K_2 = 0.5K_m EM_i = \frac{[M_2L][Lip]}{[ML][M]} \qquad \text{Eq. 4}$$

$$K_3 = K_c = \frac{[C][Lip]}{[C_0][M]} \qquad \text{Eq. S52}$$

$$K_4 = 2K_m M_f = \frac{[CL]}{[C][L]} \qquad \text{Eq. 5}$$

$$K_5 = 0.5K_m K_C M_f EM_C = \frac{[C_2L][Lip]}{[M][CL]} \qquad \text{Eq. 7}$$

The equations for the alternative routes leading to the formation of *C***L** and C_2**L** as depicted in Supplementary Figure S4 can be written as a function the equilibrium constants K_1 to K_5 as:

$$K_6 = \frac{K_3 K_4}{K_1} = K_C M_f = \frac{[CL][Lip]}{[C_0]ML} \qquad \text{Eq. S53}$$

$$K_7 = \frac{K_5}{K_3} = 0.5K_m M_f EM_C = \frac{[C_2L][C_0]}{[C][CL]} \qquad \text{Eq. S54}$$

$$K_8 = \frac{K_5 K_4}{K_1} = 0.5K_m K_C {M_f}^2 EM_C \qquad \text{Eq. S55}$$

$$K_9 = \frac{K_5 K_4 K_3}{K_2 K_1} = (K_C M_f)^2 \frac{EM_C}{EM_i} = \frac{[C_2L][Lip]}{[C_0][M_2L]} \qquad \text{Eq. S56}$$

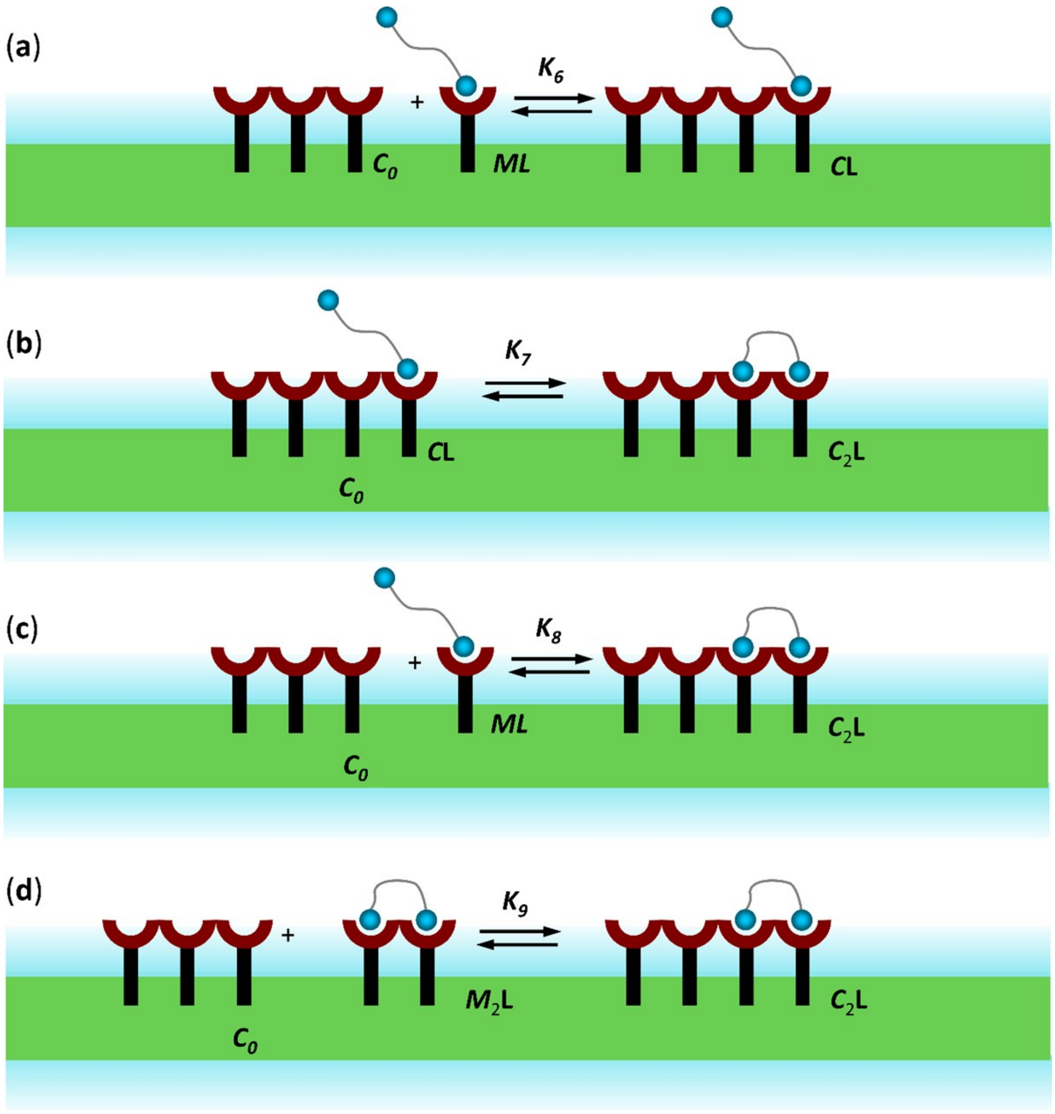

Supplementary Fig. S4. (a)-(d) Routes for the formation of species C**L** and C_2**L** in addition to the ones discussed in the running text and shown in Fig. 3.

Estimating the value of K_i in temperature stage 2: K_i as a function of the properties of the membrane-water interface.

Changes in the Soret band of the UV-Visible spectrum of **1Ch** in stage 1, in the absence of ligand, are attributed to a solvatochromic shift due to changes in the properties of the lipid-water interface. These absorbance changes correlate very well with the increase in the affinity of the ligand for the interface (Supplementary Fig. S5).

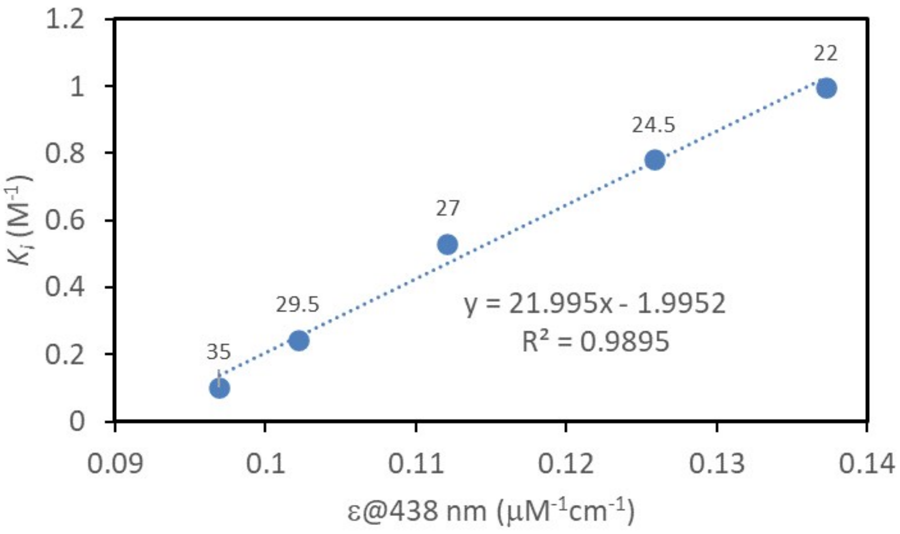

Supplementary Fig. S5. Correlation between Ki and the extinction coefficient of the ligand free monomeric receptor *M* in the membrane. The data labels are the temperature of each data point in degrees Celsius.

It is therefore reasonable to attribute the changes in K_i to the same changes in the lipid membrane interface. To account for these changes, we formulate a hypothetical equilibrium between two types of membrane interface, I_a and I_b, whose change in character is centred at the main lipid transition temperature (that is, at T_m $[I_a] = [I_b]$). The fraction of the high temperature interface population, x_{Ia}, can be written as:

$$x_{Ia} = \left(1 + e^{\frac{\Delta H_i}{R}\left(\frac{1}{T_m} - \frac{1}{t + 273}\right)}\right)^{-1} \qquad \text{Eq. S57}$$

Equation S56 is derived from the van 't Hoff equation for the change of state.[24] ΔH_i is the enthalpy associated with the changes in solvation in the membrane interface, T_m is the main lipid transition temperature in Kelvin, and t is the temperature in Celsius.

Since no lipid binding to the interface is observed at the highest temperature points (i.e. above 35 °C) we can further assume that the ligand only binds to the low-temperature interface state, I_b. The constants calculated at single temperature points, K_i , can thus be written as a function of the interface composition:

$$K_i = K_{i,0}(1 - x_{Ia}) \quad \text{Eq. S58}$$

Where $K_{i,0}$ is the intrinsic constant for the binding to the low temperature interface state. We fit the values of K_i determined for temperature stage 1 to the system composed of equations S45 and S46, and enter T_m as a known value, which was obtained from the fitting of the clustering of **1Ch** *vs* temperature (Supplementary Fig. S6). The value of ΔH_i obtained from the fitting allow us to estimate K_i at the temperature points above T_m (Supplementary Table S4).

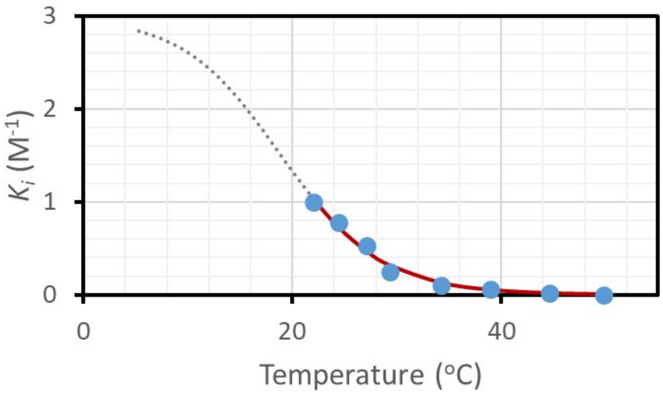

Supplementary Fig. S6. Changes in K_i with the temperature in temperature stage 1 (blue circles) and fit to the model described by equations S57 and S58 (red line). The dotted grey line is the extrapolation of the model function to temperatures in temperature stage 2 (i.e. above the T_m that gives rise to values of K_i shown in Supplementary Table S4).

Supplementary Table S4. Values of K_i in stage 2 extrapolated from the fitting of the experimental values of K_i determined at temperature stage 1, using the model composed of equations S57and S58. These values are subjected to a moderate error (25% measured as twice the standard deviation of the fit).

t (°C)	19.5	17.1	14.7	12.3	10	7.6	5.2
K_i (M^{-1})	1.4	1.8	2.1	2.4	2.6	2.7	2.8
ΔH_i (kJ mol^{-1})	150						
$K_{i,0}$ (M^{-1})	3.0						

Divalent ligand binding model for stage 2

Equilibrium equations

$$K_i = \frac{[L_i]}{[L][Lip]}$$ Eq. 2

$$K_1 = 2K_m = \frac{[ML]}{[M][L](1 + K_i EM_i)}$$ Eq. 3

$$K_2 = 0.5K_m Z_i = \frac{[M_2L][Lip]}{[M][ML]}$$ Eq. 4

$$K_c = \frac{[C][Lip]}{[C_0][M]}$$ Eq. S10

$$K_4 = 2K_m M_f = \frac{[CL]}{[C][L]}$$ Eq. 5

$$K_5 = 0.5K_m K_C M_f EM_c = \frac{[C_2L][Lip]}{[M][CL]}$$ Eq. 7

$$K_{da} = \frac{[ML \cdot L_i][C_o]}{[CL][L_i]}$$ Eq. 8

Mass balances:

$$[C_0] = [C] + [CL] + 2[C_2L] + [Nuc]$$ Eq. S59

$$[1Ch] = [M] + [ML] + 2[M_2L] + [ML \cdot L_i] + [C] + [CL] + 2[C_2L]$$ Eq. S60

$$[L]_0 = [L_i] + [L]$$ Eq. S62

Lambert-Beer law:

$$\varepsilon_{app} = \frac{A_{obs}}{[1Ch]}$$ Eq. S63

$$\varepsilon_{app} = \frac{1}{[1Ch]}\varepsilon_M[M] + \varepsilon_{ML}([ML] + [ML \cdot L_i]) + \varepsilon_{M2L}[M_2L] + \varepsilon_C[C] + \varepsilon_{CL}[CL] + \varepsilon_{C2L}[C_2L]$$

Eq. S64

The apparent extinction coefficient, ε_{app}, at 4 different wavelengths was fitted to this model (Fig. 4c, Supplementary Fig. S7). See methods for details on the fitting procedure. See Supplementary Table S5 for values of K_{da} at each temperature point analysed.

In the fitting, an important aspect to consider is the value of the nucleus concentration [*Nuc*]. For a highly cooperative assembly, the concentration of nucleus is much smaller than that of the assembly. A mathematically simple way to account for this phenomenon is to define [*Nuc*] as a small fraction of the total receptor. If it is small enough, the exact value does not have a measurable impact in the speciation, and thus the fitting, and can be assumed to be a constant.[21] In our experiments we have empirically found that for any value of [*Nuc*] less than 0.01% of the total amount of [**1Ch**], no measurable difference in the behaviour or the system is observed. We do, therefore, define the concentration of nucleus [*Nuc*] as:

$$[Nuc] = \frac{[1Ch]}{10000} \qquad \text{Eq. S65}$$

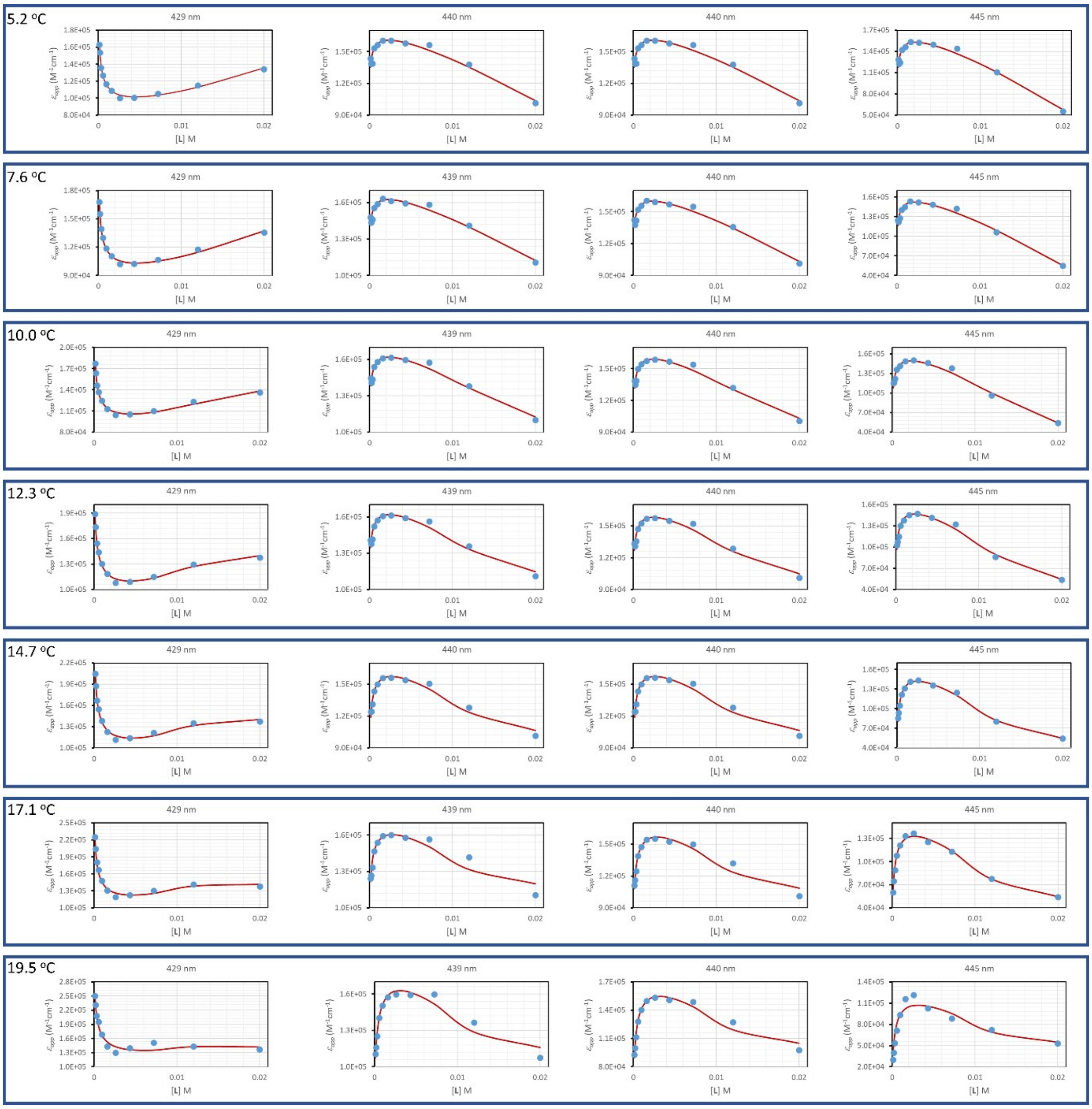

Supplementary Fig. S7. Values of ε_{app} for samples of **1Ch** in DMPC vesicles (r_{1Ch} = 0.01, [**1Ch**] = 2x10^{-6} M) at increasing concentrations of ligand **L** (solid circles) and the fitting to the temperature stage 2 model (solid red line).

Supplementary Table S5. Values of K_{da} obtained from the fitting at each temperature point in temperature stage 2. These values are subject to a moderate error (25% measured as twice the standard deviation of the fit)

t (ºC)	5.2	7.6	10	12.3	14.7	17.1	19.5
K_{da}	0.097	0.11	0.14	0.19	0.26	0.33	0.48

Disassembly of clusters upon ligand saturation

The first step of disassembly is the saturation of clustered receptor *C* with ligand (Supplementary Fig. S8a):

$$K_{10} = \frac{K_4 K_3}{K_5} = 4EM_c = \frac{[CL]^2}{[C_2L][L][C_o]} \qquad \text{Eq. S66}$$

This is followed by the disassembly of cluster *C*L by the action of membrane-partitioned ligand (Supplementary Fig. S8b):

$$K_{11} = K_{da} = \frac{[ML \cdot L_i][C_o]}{[CL][L_i]} \qquad \text{Eq. S67}$$

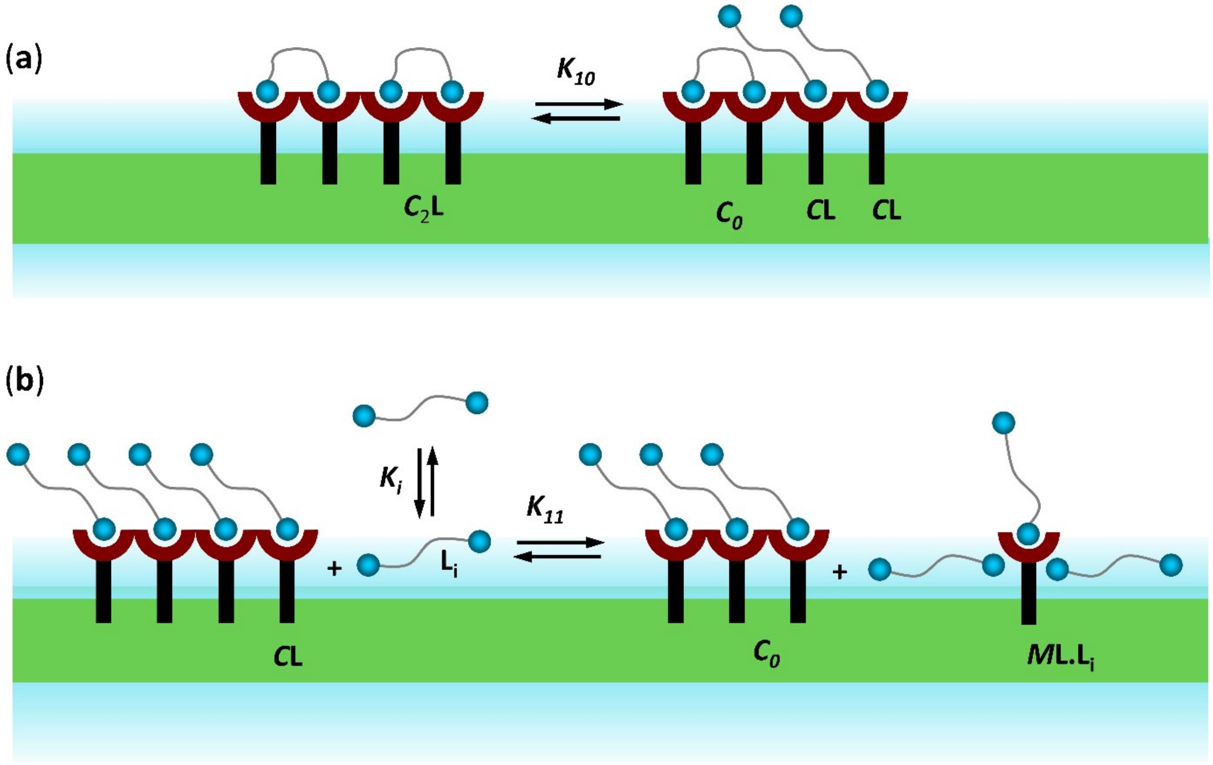

Supplementary Fig. S8. (a)-(b) Equilibria that link the most stable form of the cluster (C_2**L**) with the species that is dominant at excess ligand concentration (*M***L.L**$_i$).

In-membrane solubility on a ligand-saturated membrane and correlation with the in-membrane solubility in the absence of the ligand.

In excess of ligand the dominant form of the cluster is *C***L**. We can therefore assume that:

$$[C_o] = [CL] \qquad \text{Eq. S68}$$

Under these conditions, Eq. 8 can be simplified as:

$$K_{da} = \frac{[ML \cdot L_i]}{[L_i]}$$ Eq. S69

That is, K_{da} is the maximum in-membrane concentration of the complex *M***L** on a ligand saturated membrane.

We can determine the increase in in-membrane solubility of the receptor, in a ligand saturated membrane, by comparing with $r_{M,max}$, (that is, the inverse of K_C) in the absence of ligand. A graphical representation of K_{da} *vs* $r_{M,max}$ shows that there is a linear correlation (Supplementary Fig. S9). From the slope of the trendline we estimate the increase in in-membrane solubility to be 45-fold.

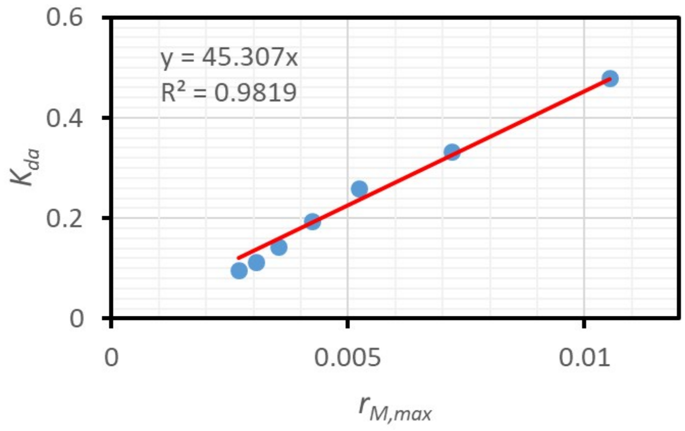

Supplementary Fig. S9. Representation of the values of K_{da} *vs* $r_{M,max}$ at each of the temperature points in temperature stage 2 (blue circles), fit to a straight line.

Derivation of the van 't Hoff equation for lipid phase change.

The change of lipid phase can be written as an equilibrium between the phases *G* and L_d as:

$$K_t = \frac{[L_d]}{[G]}$$ Eq. S70

Which, when written as a function of the mol fraction of the L_d phase, x_{Ld}, can be written as:

$$K_t = \frac{x_{Ld}}{1 - x_{Ld}} \qquad \text{Eq. S71}$$

Furthermore, K_t can be written as a function of the temperature using the integrated form of the van 't Hoff equation:[25]

$$lnK_t = - \frac{\Delta H_m}{R}\left(\frac{1}{T_m} - \frac{1}{t + 273}\right) \qquad \text{Eq. S72}$$

Where ΔH_m is the enthalpy of phase transition, T_m is the temperature of phase transition in Kelvin, and t the temperature in degrees Celsius. Combining with Eq. S71, and re-arranging, gives:

$$x_{Ld} = \left(1 + e^{\frac{\Delta H_m}{R}\left(\frac{1}{T_m} - \frac{1}{t + 273}\right)}\right)^{-1} \qquad \text{Eq. 11}$$

Thermodynamic parameters for the lateral assembly of the receptor into clusters *C* at temperature stage 2

The van 't Hoff equation for the dependence of K_C with the temperature is:

$$K_C = e^{\left(-\frac{\Delta H_c}{RT} + \frac{\Delta S_c}{R}\right)} \qquad \text{Eq. 12}$$

which in the classic linearized form can be written as:

$$lnK_C = - \frac{\Delta H_c}{RT} + \frac{\Delta S_c}{R} \qquad \text{Eq. S73}$$

Plotting lnK_C *vs* $1/T$, ΔH_C can be calculated from the slope of the liner fit and ΔS_C from the intercept (Supplementary Fig. S10, Table 1).

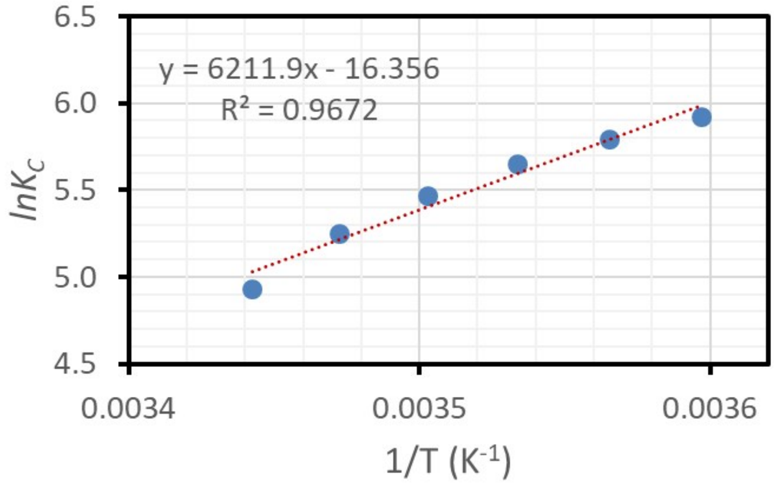

Supplementary Fig. S10. Van 't Hoff plot of the values of K_C in temperature stage 2. The slope and intercept are shown in the graph, as well as the linear correlation coefficient. See Table 1 for values of ΔH_c and ΔS_c.

Model of in-membrane receptor assembly with temperature dependence

1. Van 't Hoff equations

Main transition:

$$x_{Ld} = \left(1 + e^{\frac{\Delta H_m}{R}\left(\frac{1}{T_m} - \frac{1}{t + 273}\right)}\right)^{-1} \quad \text{Eq. 11}$$

Dependence of interface property with the temperature (ΔH_i was obtained from the fitting of K_i, see Supplementary Fig. S6 and Table S4):

$$x_{Ia} = \left(1 + e^{\frac{\Delta H_i}{R}\left(\frac{1}{T_m} - \frac{1}{t + 273}\right)}\right)^{-1} \quad \text{Eq. S57}$$

Dependence of the clustering constant with the temperature:

$$K_C = e^{\left(-\frac{\Delta H_c}{RT} + \frac{\Delta S_c}{R}\right)} \quad \text{Eq. 12}$$

2. Mass balances

$$[Lip]_i = x_{Ld}[Lip] + \frac{1 - x_{Ld}}{n_C}[Lip] \quad \text{Eq. 10}$$

$$[C_0] = [C] + [Nuc] \quad \text{Eq. S9}$$

$$[1Ch] = [M] + [C]$$ Eq. S3

3. Equilibrium equations

$$K_c = \frac{[C][Lip]_i}{[C_0][M]}$$ Eq. S10

4. Lambert-Beer law

$$\varepsilon_{app} = \frac{A_{obs}}{[1Ch]}$$ Eq. S63

$$\varepsilon_{app} = \frac{1}{[1Ch]}(\varepsilon_M[M] + \varepsilon_C[C])$$ Eq. S74

$$\varepsilon_M = \varepsilon_{Ma}x_{Ia} + \varepsilon_{Mb}(1 - x_{Ia})$$ Eq. S75

Eq. S75 accounts for the spectral changes attributed to the receptor anchored in each form of the interface, with ε_{Ma} and ε_{Mb} the extinction coefficients for pure I_a and I_b, respectively.

The apparent extinction coefficient, ε_{app}, at 445 nm (where the band of the assembled receptor *C* is dominant) was fitted to this model (Supplementary Fig. S11). See methods for details on the fitting procedure and Table 1 for thermodynamic parameters.

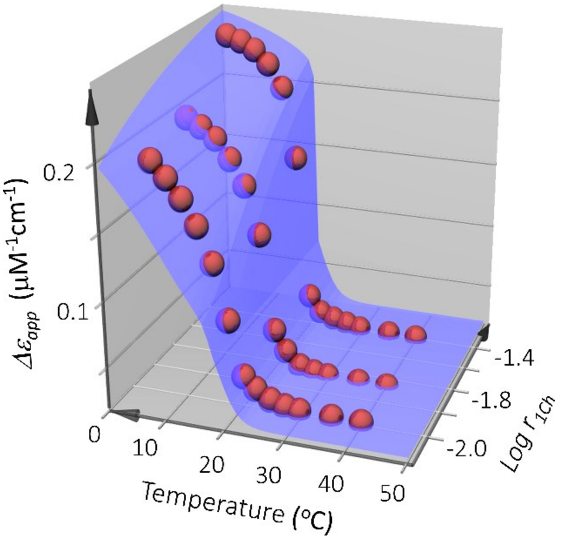

Supplementary Fig. S11. ΔE_{app} values at 445 nm extracted from the UV-visible spectrum for samples of membrane embedded **1Ch** at different values of in-membrane concentration and temperatures

(red spheres). These were fit to the in-membrane assembly with temperature dependence assembly model (blue surface). See Methods for experimental details.

Global model of in-membrane receptor assembly and ligand binding with temperature dependence

1. Van 't Hoff equations

Main transition:

$$x_{Ld} = \left(1 + e^{\frac{\Delta H_m}{R}\left(\frac{1}{T_m} - \frac{1}{t + 273}\right)}\right)^{-1}$$ Eq. 11

Interface property dependence with the temperature:

$$x_{Ia} = \left(1 + e^{\frac{\Delta H_i}{R}\left(\frac{1}{T_m} - \frac{1}{t + 273}\right)}\right)^{-1}$$ Eq. S57

Dependence of the clustering constant with the temperature:

$$K_C = e^{\left(-\frac{\Delta H_C}{RT} + \frac{\Delta S_C}{R}\right)}$$ Eq. 12

2. Mass balances

$$[Lip]_i = x_{Ld}[Lip] + \frac{1 - x_{Ld}}{n_C}[Lip]$$ Eq. 10

$$[C_0] = [C] + [CL] + 2[C_2L] + [Nuc]$$ Eq.S59

$$[1Ch] = [M] + [ML] + 2[M_2L] + [ML \cdot L_i] + [C] + [CL] + 2[C_2L]$$ Eq. S60

$$[L]_0 = [L_i] + [L]$$ Eq. S62

3. Equilibrium equations

Ligand membrane partition

$$K_{i,0} = \frac{[L_i]}{[L][Lip](1 - x_{Ia})}$$ Eq. S76

Receptor assembly and disassembly

$$K_c = \frac{[C][Lip]_i}{[C_0][M]}$$ Eq. S77

$$\frac{45}{K_c} = \frac{[ML \cdot L_i][C_o]}{[CL][L_i]}$$ Eq. S78

Receptor ligand binding

$$2K_m = \frac{[ML]}{[M][L](1 + K_i EM_i)}$$ Eq. S79

$$0.5 K_m Z_i = \frac{[M_2L][[Lip]_i}{[M][ML]}$$ Eq. S80

$$2K_m M_f = \frac{[CL]}{[C][L]}$$ Eq. S81

$$0.5 K_m K_C M_f EM_c = \frac{[C_2L][Lip]_i}{[M][CL]}$$ Eq. S82

4. Lambert-Beer law

$$\varepsilon_{app} = \frac{A_{obs}}{[1Ch]}$$ Eq. S63

$$\varepsilon_{app} = \frac{1}{[1Ch]} \varepsilon_M[M] + \varepsilon_{ML}([ML] + [ML \cdot L_i] +) + \varepsilon_{M2L}[M_2L] + \varepsilon_C[C] + \varepsilon_{CL}[CL] + \varepsilon_{C2L}[C_2L]$$

Eq. S83

$$\varepsilon_M = \varepsilon_{Ma} x_{Ia} + \varepsilon_{Mb}(1 - x_{Ia})$$ Eq. S75

Equation S76 is obtained by combining Equation 2 and Equation S58. Equations S79, S80, S81 and S82 are obtained from Equations 3, 4, 5 and 7 respectively by replacing the total concentration of lipids [*Lip*] for the apparent one $[Lip]_i$ Equation S75 accounts for the spectral changes attributed to the receptor anchored in each form of the interface, with ε_{Ma} and ε_{Mb} the extinction coefficients for pure I_a and I_b, respectively.

The apparent extinction coefficient, ε_{app}, at 429 nm (i.e. the wavelength that experiences the largest change) was fitted to this model (Fig. 5a). See methods for details on the fitting procedure. See Table 1 for thermodynamic parameters.

Multivalent platform deployment as a function of the ligand concentration

Multivalent platform *xMV* can be written as a function of the cluster forms as:

$$xMV = \frac{[C] + [CL] + 2[C_2L]}{[1Ch]}$$ Eq. S84

Except in conditions of large excess of ligand, the concentration of *C***L** is much smaller than those of C_2**L** and *C*. Eq. S84 can thus be written as:

$$xMV = \frac{[C] + 2[C_2L]}{[1Ch]}$$ Eq. S85

In these conditions, the following mass balance applies:

$$[1Ch] = [M]_{max} + [C] + 2[C_2L]$$ Eq. S86

Which substituted into equation S85 yields:

$$xMV = \frac{[1Ch] - [M]_{max}}{[1Ch]}$$ Eq. S87

Rearrangement as a function of the in-membrane solubilities gives:

$$xMV = 1 - \frac{r_{M_{max}}}{r_{1Ch}}$$ Eq. S88

r_{Mmax} is the inverse of the clustering constant for these experimental conditions, K_C'. Re-writing Equation S88 to account for this, we have:

$$xMV = 1 - \frac{1}{r_{1Ch}K_C'}$$ Eq. S89

Written as a function of the species present, K_C' is:

$$K_c' = \frac{([C] + 2[C_2L])[Lip]}{[C_0][M]}$$ Eq. S90

Which can be arranged as:

$$K_c' = K_c + \frac{2[C_2L][Lip]}{[C_0][M]}$$ Eq. S91

The formation of C_2**L** can be written as a function of *M* and **L** as:

$$(K_mM_fK_c)^2EM_c = \frac{[C_2L][Lip]^2}{[C_0][M]^2[L]}$$ Eq. S92

In the hypothetical case where the only form of cluster is C_2**L**, we have that:

$$2[C_2L] = [C_0]$$ Eq. S93

which substituted into equation S92 yields:

$$(K_m M_f K_c)^2 EM_c [L] = \frac{[Lip]^2}{2[M]_{max,L}{}^2}$$ Eq. S94

Where $[M]_{max,L}$ is the maximum concentration of monomer present when C_2L is the dominant species. The corresponding clustering constant $K_{C,L}$ is:

$$K_{C,L} = \frac{[Lip]}{[M]_{max,L}}$$ Eq. S95

Which combined with equation S94 is:

$$K_{C,L} = K_m M_f K_c \sqrt[2]{2[L]EM_c}$$ Eq. S96

On the other hand, combining equations S91 and S95 with equation S93 we have:

$$K_c' = K_c + K_{C,L}$$ Eq. S97

Substituting in equation S96 we have:

$$K_c' = K_c(1 + K_m M_f \sqrt[2]{2[L]EM_c})$$ Eq. S98

By substituting equation S98 into equation S89:

$$xMV = 1 - \frac{1}{r_{1Ch} K_c (1 + K_m M_f \sqrt[2]{2[L]EM_c})}$$ Eq. S99

Which, in the general case of *n* binding sites, becomes:

$$xMV = 1 - \frac{1}{r_{1Ch} K_c (1 + K_m M_f \sqrt[n]{n[L]EM_c{}^{n-1}})}$$ Eq. 13

If r_{1Ch} equals r_{Mmax}, we can simplify to:

$$xMV = 1 - \frac{1}{1 + K_m M_f \sqrt[n]{n[L]EM_c{}^{n-1}}}$$ Eq. 14

At 50% signal deployment xMv = 0.5. In these conditions we have that:

$$\frac{1}{2} = \frac{1}{1 + K_m M_f \sqrt[n]{n[L]EM_c{}^{n-1}}}$$ Eq. S100

Which can be re-arranged as:

$$K_m M_f \sqrt[n]{n[L]EM_c{}^{n-1}} = 1$$ Eq. S101

And isolating [L] is:

$$[L] = n^{-1}(K_m M_f)^{-n} EM_c{}^{1-n}$$ Eq. 15
